# Opposite Roles of IL-32α Versus IL-32β/γ Isoforms in Promoting Monocyte-Derived Osteoblast/Osteoclast Differentiation and Vascular Calcification in People with HIV

**DOI:** 10.3390/cells14070481

**Published:** 2025-03-22

**Authors:** Hardik Ramani, Aurélie Cleret-Buhot, Mohamed Sylla, Rémi Bunet, Florent Bertrand, Marc-Messier Peet, Carl Chartrand-Lefebvre, Benoit Trottier, Réjean Thomas, Jean-Pierre Routy, Claude Fortin, Valérie Martel-Laferrière, Manel Sadouni, Guy Cloutier, Louise Allard, Jorge R. Kizer, Nicolas Chomont, Petronela Ancuta, David B. Hanna, Robert C. Kaplan, Mohammad-Ali Jenabian, Alan L. Landay, Madeleine Durand, Mohamed El-Far, Cécile L. Tremblay

**Affiliations:** 1 Département de Microbiologie, Infectiologie et Immunologie, Faculté de Médecine, Université de Montréal, Montréal, QC H3C 3J7, Canada; hardik.ramani@umontreal.ca (H.R.); remi.bunet@umontreal.ca (R.B.); florent.bertrand@umontreal.ca (F.B.); claude.fortin.med@ssss.gouv.qc.ca (C.F.); valerie.martel-laferriere.med@ssss.gouv.qc.ca (V.M.-L.); nicolas.chomont@umontreal.ca (N.C.); petronela.ancuta@umontreal.ca (P.A.); 2 Centre de Recherche du Centre Hospitalier de l’Université de Montréal (CRCHUM), Montréal, QC H2X 0A9, Canada; aurelie.cleret-buhot.chum@ssss.gouv.qc.ca (A.C.-B.); syllmoh@yahoo.fr (M.S.); marc.messier-peet.chum@ssss.gouv.qc.ca (M.-M.P.); carl.chartrand-lefebvre@umontreal.ca (C.C.-L.); sadounamine@outlook.fr (M.S.); guy.cloutier@umontreal.ca (G.C.); louise.allard.chum@ssss.gouv.qc.ca (L.A.); madeleine.durand@umontreal.ca (M.D.); 3 Cellular Imaging Core Facility, Centre de Recherche du Centre Hospitalier de l’Université de Montréal (CRCHUM), Montréal, QC H2X 0A9, Canada; 4 Département de Radiologie, Radio-Oncologie et Médecine Nucléaire, Faculté de Médecine, Université de Montréal, Montréal, QC H3T 1J4, Canada; 5 Centre de Médecine Urbaine du Quartier Latin, Montréal, QC H2L 0B1, Canada; bentrotte@gmail.com; 6 Clinique Médicale l’Actuel, Montréal, QC H2L 4P9, Canada; rejean.thomas@lactuel.ca; 7 Research Institute of McGill University Health Centre, Montréal, QC H4A 3J1, Canada; jean-pierre.routy@mcgill.ca; 8 Cardiology Section, San Francisco Veterans Affairs Health Care System, and Department of Medicine, Epidemiology and Biostatistics, the University of California San Francisco, San Francisco, CA 94121, USA; jorge.kizer@ucsf.edu; 9 Department of Epidemiology and Population Health, Albert Einstein College of Medicine, Bronx, NY 10461, USA; david.hanna@einsteinmed.edu (D.B.H.); robert.kaplan@einsteinmed.edu (R.C.K.); 10 Divsion of Public Health Sciences, Fred Hutchinson Cancer Research Center, Seattle, WA 98109, USA; 11 Department of Biological Sciences, Université du Québec à Montréal, Montréal, QC H2X 1Y4, Canada; jenabian.mohammad-ali@uqam.ca; 12 Department of Internal Medicine and Microbiology and Immunology, University of Texas, Medical Branch, Austin, TX 77555, USA; allanday@utmb.edu; 13 Département de Médecine, Faculté de Médecine, Université de Montréal, Montreal, QC H3T 1J4, Canada

**Keywords:** HIV, inflammation, IL-32, cardiovascular diseases, arterial calcification, atherosclerosis, osteoclasts, osteoblasts, osteoprotegerin, TGF-β

## Abstract

People with HIV (PWH) have an increased risk of developing cardiovascular disease (CVD). Our recent data demonstrated that the multi-isoform proinflammatory cytokine IL-32 is upregulated in PWH and is associated with arterial stiffness and subclinical atherosclerosis. However, the mechanisms by which IL-32 contributes to the pathogenesis of these diseases remain unclear. Here, we show that while the less expressed IL-32α isoform induces the differentiation of human classical monocytes into the calcium-resorbing osteoclast cells, the dominantly expressed isoforms IL-32β and IL-32γ suppress this function through the inhibition of TGF-β and induce the differentiation of monocytes into the calcium-depositing osteocalcin+ osteoblasts. These results aligned with the increase in plasma levels of osteoprotegerin, a biomarker of vascular calcification, and its association with the presence of coronary artery subclinical atherosclerosis and calcium score in PWH. These findings support a novel role for the proinflammatory cytokine IL-32 in the pathophysiology of CVD by increasing vascular calcification in PWH.

## 1. Introduction

Antiretroviral therapy (ART) has remarkably reduced AIDS-associated mortality and increased life expectancy in people with HIV (PWH) [1,2]. However, a gap in life expectancy without non-AIDS comorbidities such as cardiovascular disease (CVD), cancers, diabetes, and renal disease still exists in PWH compared to the general population, and this gap has not closed over the past 20 years [3]. A better understanding of the pathogenesis of these comorbidities by focusing on common causes/drivers represents a research priority to improve risk stratification and prevention. In this regard, a major and common feature for these comorbidities is chronic inflammation, which is driven by a combination of HIV-mediated factors and by the aging process itself, as reflected by the upregulation of multiple inflammatory factors, including TNF-α, IL-1β, IL-6, D-dimers, and high-sensitivity C-reactive protein (hsCRP) [4,5]. Many of these inflammatory mediators have been shown to be involved in immune cell activation, endothelium dysfunction, and the induction of other inflammatory cytokines leading to increased risk for the development of non-AIDS comorbidities including CVD, a leading cause of mortality, as well as osteoporosis [1,3,6,7,8,9,10,11].

While the latter conditions have been traditionally regarded as unrelated, recent research suggests an association between bone and cardiovascular diseases, as people with osteoporosis are at elevated risk of developing CVD compared to those with normal bone mass [12,13,14,15,16]. Additionally, recent studies revealed that the processes of vascular calcification and decalcification employ similar cells to those involved in bone homeostasis, mainly osteoblasts (cells involved in calcium deposition) and osteoclasts (cells involved in calcium resorption) [13,17,18]. While the mechanisms governing the differentiation and activation of these cell types are closely coupled to ensure bone homeostasis and to potentially ensure normal vascular physiology, inflammatory conditions and the upregulation of inflammatory cytokines such as TNF-α, IL-1, and IL-6 are known to interfere and perturb the fine balance between these cells and, consequently, their respective functions [19,20,21]. In PWH, many of these cytokines are known to be chronically upregulated even under ART and to be associated with an increased risk of CVD [4,5,22,23]. However, how these cytokines are upregulated and whether they are mechanistically involved in the pathogenesis of both CVD and bone disease remain unknown.

We and others have previously shown that HIV infection induces the upregulation of the multi-isoform proinflammatory cytokine IL-32, and that IL-32 is associated with disease progression and CVD in PWH [24,25,26]. IL-32 in turn induces the expression of multiple other inflammatory mediators, including TNF-α, IL-1β, IL-6, and IL-18, which likely positions IL-32 upstream of a complex inflammatory network linked with different comorbidities. At the mechanistic level, our studies showed that the dominantly expressed IL-32 isoforms β and γ may contribute to subclinical atherosclerosis by inducing M1 macrophage differentiation and downregulation of the atheroprotecting TRAIL protein [25]. These isoforms may also induce accentuated recruitment of monocytes/macrophages to the coronary artery endothelium, which contributes to endothelial cell dysfunction and arterial stiffness [27]. Notably, there is a vicious cycle between arterial stiffness and arterial calcification where they reinforce each other [28], a mechanism involving osteoblast cell functions [29,30]. Interestingly, IL-32 isoforms were shown to be involved in the differentiation of osteoblasts and osteoclasts. Studies in a murine model showed that the largest and most inflammatory IL-32 isoform γ promotes osteoblast formation synergistically with RANKL (receptor activator of nuclear factor kappa beta (NFkB ligand) [31]. In humans, IL-32γ increases osteoblast differentiation in people with conditions of abnormal bone formation (ankylosing spondylitis) and is associated with the pathogenesis of disease [32]. In contrast, the less expressed and potentially anti-inflammatory isoform IL-32α was shown to induce osteoclastogenesis synergistically with macrophage colony-stimulating factor (M-CSF), RANKL, and/or IL-17A secreted by precursors cells generated from peripheral blood mononuclear cells (PBMCs) [33,34,35].

Given the upregulation of IL-32 isoforms in PWH with arterial stiffness and CVD, we aimed in the current study to investigate the impact of these isoforms on the differentiation of human monocytes/macrophages to either osteoclasts or osteoblasts, cells involved in the crosstalk between heart and bone diseases. We demonstrate that the dominantly expressed isoforms IL-32β and IL-32γ induce the differentiation of classical monocytes into osteocalcin+ osteoblast-like cells in a mechanism involving the inhibition of TGF-β. These data suggest a role for IL-32 in vascular calcification in PWH. This was further supported by our observations that PWH in whom we recently demonstrated the chronic upregulation of IL-32 and its association with arterial stiffness and subclinical atherosclerosis have higher plasma levels of the vascular calcification marker osteoprotegerin when compared to the general population. Osteoprotegerin plasma levels in these study participants were associated with the calcium score, the presence of coronary artery atherosclerotic plaques, and the presence of arterial stenosis.

## 2. Materials and Methods

### 2.1. Study Population

Plasma and peripheral blood mononuclear cells (PBMCs) were collected from PWH and controls participating in the Canadian Cohort of HIV and Aging Study (CHACS), a multi-centered prospective cohort study aiming to determine immune correlates associated with successful aging with HIV [36]. Inclusion criteria were as follows: people living with and without HIV had to be aged ≥ 40 years, with a life expectancy of at least 1 year, and ability to provide informed consent in French or English. PWH aged < 40 years were also eligible if they met the criteria of having been living with HIV for at least 15 years. All participants provided written informed consent. Demographics and clinical characteristics of the study participants (PWH, n = 168, and HIV-negative controls, n = 84) are summarized in Table 1 and Table 2. The average age of PWH was 56 ± 6.8 years compared to the HIV-negative group (56.4 ± 8.7 years). The calcium score and subclinical atherosclerosis, defined in the study participants by the presence of atherosclerotic plaque (plaque+) in the coronary arteries, were measured using a cardiac computed tomography (CT) scan with injection of contrast media [37].

### 2.2. Isolation, Culture, and Stimulation of Primary Monocytes

CD14+ monocytes were isolated from total PBMCs from uninfected HIV-negative controls by negative magnetic selection using EasySep Human CD14+ isolation kit from STEMCELL as per instructions of the manufacturer (full description and reference numbers for all materials used in the current work can be found in Supplemental Table S1). The purity of CD14+ monocyte cells was typically >98% following two cycles of negative isolation as determined by surface staining and performing flow cytometry analysis, as shown in Figure S1. Isolated monocytes were resuspended in complete α-MEM medium containing 2 mM of L-glutamine, 10% fetal bovine serum (FBS), 100 IU/mL of penicillin, 100 mg/mL of streptomycin, and Polymyxin-B. One million cells were cultured on sterilized cover slip #1.5 with 70% ethanol in 24-well cell culture plate. Cells were stimulated with recombinant human macrophage colony stimulating factor (M-CSF) (25 ng/mL); recombinant human sRANKL (30 ng/mL); and 500 ng/mL of recombinant IL-32 isoforms IL-32α, IL-32β, or IL-32γ as we previously described [25], either individually or in combination for 21 days. Half culture medium was replaced with fresh medium and stimulant(s) every 3 days, and the cultures were maintained for 21 days. Differentiated cells were evaluated by immunofluorescence imaging.

### 2.3. Immunofluorescence Staining

Cells were washed three times with phosphate buffered saline (PBS) without calcium or magnesium in 24-well cell culture plates. Cells were then fixed with 4% paraformaldehyde (PFA) in PBS for each condition and incubated for 10–15 min at 23 °C. Cells were washed three times with PBS before being permeabilized with 0.2% Triton-X-100 detergent in PBS- for 3 min. Following being washed three times with PBS, cells were blocked for 10–15 min with 10% donkey serum in PBS. Anti-tartrate-resistant acid phosphatase (TRAP/ACP5) for TRACP, Alexa Fluor™ 647 Phalloidin for F-Actin, and/or Anti-Osteocalcin Polyclonal Antibody for osteocalcin were used at a 1:100 dilution in PBS- containing 3% donkey serum. Cells were then incubated overnight at 4 °C in the dark with primary antibodies followed by being washed three times with PBS. Cells were then incubated with the secondary antibodies (diluted in PBS- containing 3% donkey serum) for one hour at 23 °C in the dark. Donkey anti-mouse IgG (H + L) highly cross-adsorbed secondary antibody, Alexa Fluor 488- (1:400), and anti-rabbit IgG (H + L), F(ab’)2 Fragment, Alexa Fluor^®^ 555 conjugate were used as secondary antibodies for TRACP and osteocalcin, respectively. Nuclei were stained with DAPI (1:1000) diluted in PBS for 15 min at 23 °C. Cover slip with cells was invertedly mounted on glass slide using ProLong™ Gold antifade mountant (Invitrogen, Eugene, OR, USA). Mounted slides were kept overnight in a dry and dark place. Multiplex spinning-disk confocal imaging was used to identify and analyze the differentiated cells.

### 2.4. Cell Culture, Stimulation, and Osteogenic Differentiation of Human Mesenchymal Stem Cells (hMSC)

Human mesenchymal stem cells (hMSC) were thawed and sub-cultured using mesenchymal stem cell growth medium as per supplier’s instructions. Cells were maintained using MSCGM™ in black clear bottom 96-well plate. Cells were allowed to adhere to the culture surface for 4 to 24 h in MSCGM™ at 37 °C in a humidified incubator with 5% CO_2_. hMSCs were stimulated with individual IL-32 isoforms at 500 ng/mL in MSCGM, as shown in Figure S2. As a positive control, osteogenic differentiation of hMSCs was carried out by using differentiation media osteogenic BulletKit™ (LONZA, Walkersville, MD, USA) as per manufacturer’s instructions. Non-induced control cells were maintained by subculturing using MSCGM™. Cells were maintained for 3 weeks by completely replacing the medium with fresh osteogenesis induction medium every 4 days. Non-induced control hMSCs and IL-32 isoform-stimulated hMSCs were maintained with MSCGM™ and individual IL-32 isoform, respectively, on the same schedule. hMSCs were assessed using immunofluorescence staining involving TRAcP, F-actin, osteocalcin, and nuclei, as described above.

### 2.5. Image Acquisition and Analysis

**Figure 1:**
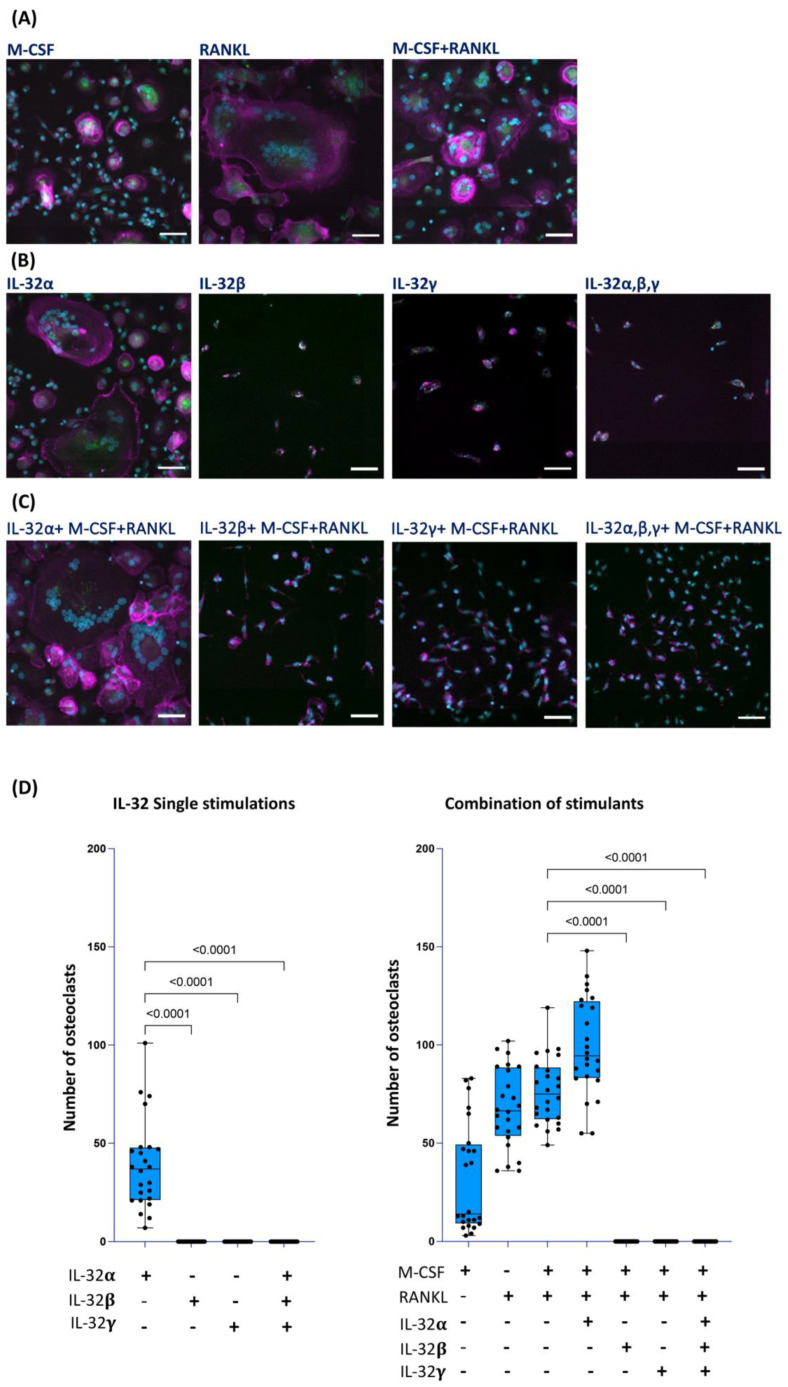
Impact of IL-32 isoforms on monocyte differentiation into osteoclasts. Representative multiplexed spinning-disk confocal (20×/0.8) images of monocytes isolated from HIV-negative cells stimulated with either (**A**) M-CSF, RANKL, or M-CSF+ RANKL; or (**B**) IL-32α, IL-32β, IL-32γ, or with combination of three isoforms; or (**C**) combinations of individual IL-32 isoforms with M-CSF + RANKL. Osteoclasts are defined as cells expressing TRACP5 (green), formation of F-actin rings (magenta), and presence of three or more nuclei (DAPI, blue) as shown in (**A**) (all panels) and (**B**,**C**) (left panels). (**D**) Analysis of osteoclast numbers from monocytes isolated from n = 6 donors and induced with individual IL-32 isoforms alone (left panel) or combined with RANKL and M-CSF (right panel). Data represent analyses by counting 4 image clusters (3 × 3 tiles per cluster) representing 36 image tiles per condition per donor using Zeiss Zen 3.2 software. Nonparametric Kruskal–Wallis test and Dunn’s subtests were performed for statistical analysis. Comparisons were carried out with respect to positive control conditions M-CSF + RANKL. Scale bars represent 50 µm.

**Figure 2:**
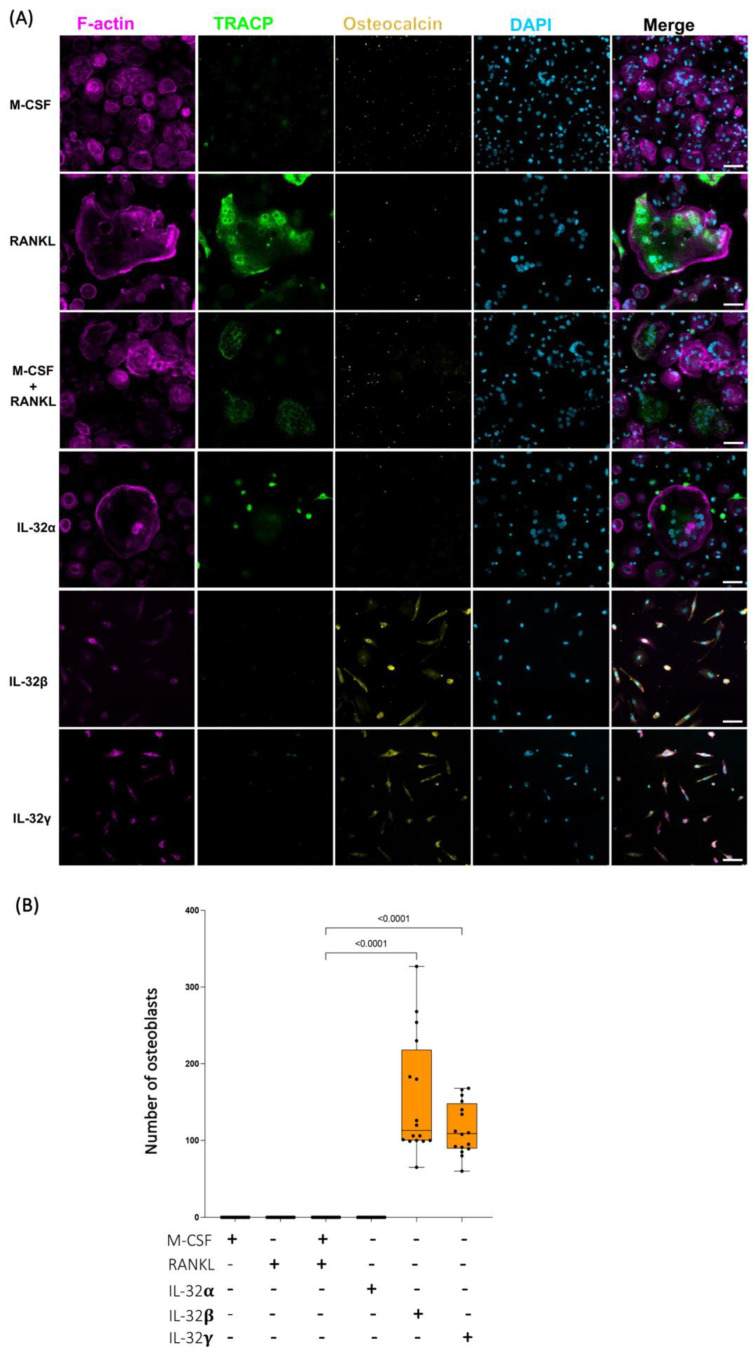
Distinct effects of IL-32 isoform IL-32α, IL-32β, and IL-32γ on differentiation of primary human monocytes into either osteoclasts or osteoblast-like cells. (**A**) Representative multiplex spinning-disk confocal (20×/0.8) images illustrating influence of individual IL-32 isoforms as well as osteoclastogenic molecules RANKL and/or M-CSF on monocyte differentiation. Osteoblast-like cells are defined as single-nucleated cells with expression of osteocalcin (yellow). Single color images are used to show F-actin, TRACP5, osteocalcin, and DAPI (from left to right) followed by merged color images (right panels). (**B**) Quantification of osteocalcin+ osteoblast-like cell numbers under different stimulation conditions and counted by automated counting using FIJI ImageJ software (Fiji, ImageJ, 64-bit). Data analysis was performed on 4 image clusters (3 × 3 tiles per cluster) representing 36 image tiles per condition per donor (cells from n = 4 HIV-negative donors). Nonparametric Kruskal–Wallis test and Dunn’s subtests were performed for statistical analysis. Scale bars represent 50 µm.

**Figure 3:**
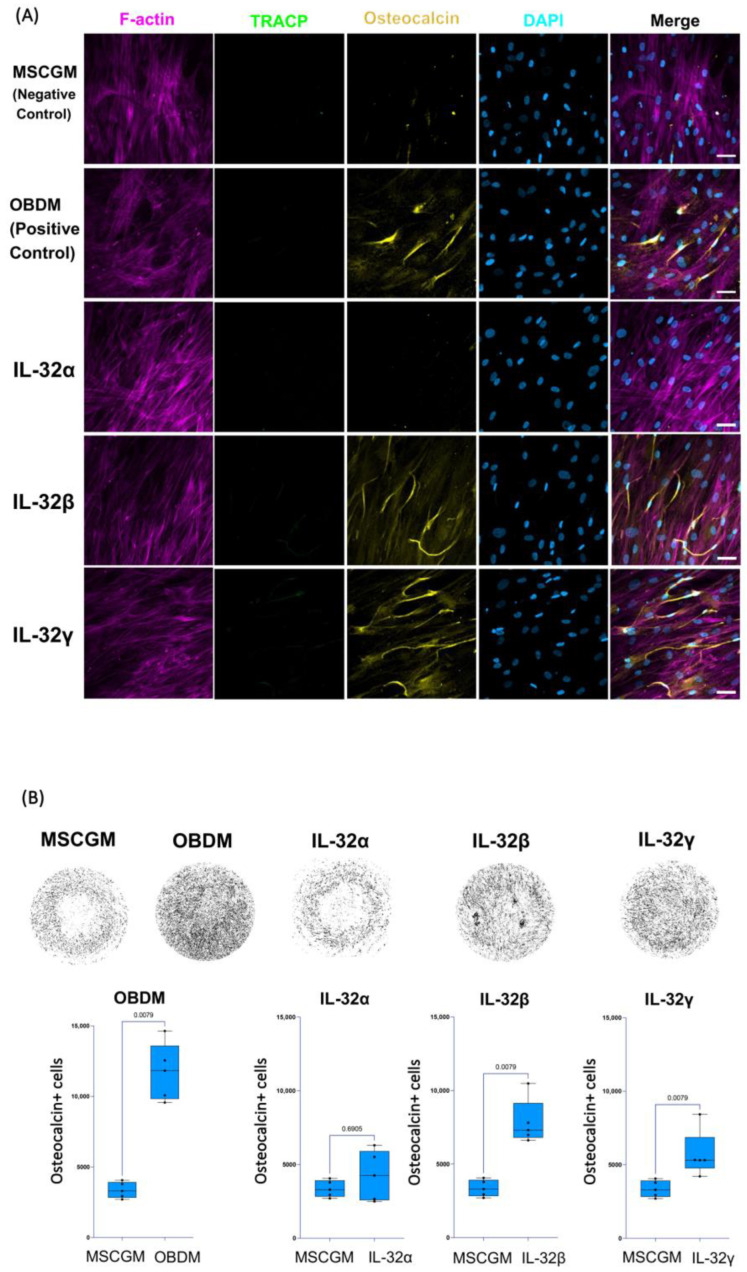
Impact of individual IL-32 isoforms IL-32α, IL-32β, and IL-32γ on differentiation of human mesenchymal stem cells (hMSCs) into osteoblasts. (**A**) Representative multiplex spinning-disk confocal (20×/0.8) images demonstrating impact of osteoblast differentiation medium/OBDM used as positive control (upper panels), IL-32 isoforms (middle panels), or negative control mesenchymal stem cell growth medium/MSCGM (lower panels) on hMSC differentiation. Single color images are used to show F-actin, TRACP5, osteocalcin, and DAPI (from left to right) followed by merged color images on far-right. Differentiated hMSC cells were identified by expression of osteocalcin. (**B**) Upper panels: representative full-well images of each condition at 10×/0.3 depicting osteocalcin expression in different conditions (dark areas indicate osteocalcin expression in cells). Lower panels: Comparing numbers of osteocalcin+ mononucleated osteoblasts from hMSCs stimulated with positive control conditions (osteoblast differentiation medium/OBDM) or with individual IL-32 isoforms compared to same negative control conditions using cell growth MSCG medium (experiments were performed simultaneously with single negative control experiment). Quantification was performed using FIJI ImageJ software, analyzing entire well for each condition using customized osteocalcin and DAPI expression measurement macro for ImageJ software (n = 5 experimental replicates). Statistical analysis was conducted using nonparametric Mann–Whitney test. Scale bars represent 50 µm.

**Figure 4:**
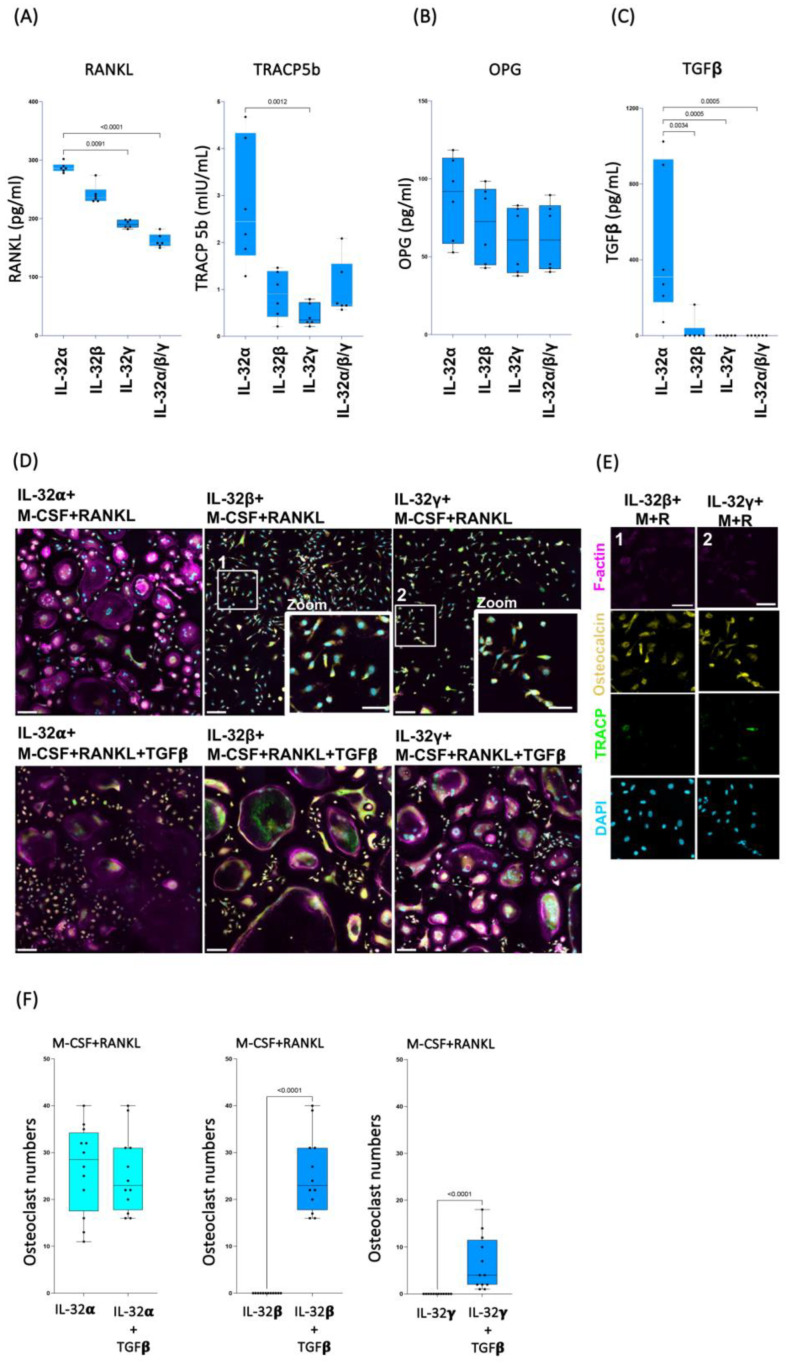
TGF-β promotes osteoclast formation in presence of IL-32β and IL-32γ. (**A**–**C**) Effect of IL-32 isoforms IL-32α, IL-32β, and IL-32γ on expression of soluble RANKL, TRACP5b, OPG, and TGF-β. Soluble proteins were measured by ELISA in supernatant of primary monocytes stimulated with IL-32 isoforms for 21 days (n = 6). Statistical analyses were performed using nonparametric Kruskal–Wallis test and Dunn’s subtest. (**D**,**E**) Representative multiplex spinning-disk confocal (20×/0.8), 3 × 3 tile images showing differentiation of monocytes into osteoclasts or osteoblasts in response to IL-32 isoforms (IL-32α, IL-32β, or IL-32γ) combined with osteoclastogenic molecules M-CSF(M)/RANKL(R) (**D**, upper panels, and **E**), and with TGF-β (**D**, lower panels). The 2 zoomed-in regions (**D**, 1 and 2) in panels show typical single-nucleated osteocalcin+ osteoblast cells induced by IL-32β and IL-32γ in absence of TGF-β. In (**E**), single-color images are used to show F-actin, osteocalcin, TRACP5, and DAPI (from top to bottom) of 2 zoomed-in regions in (**D**). (**F**) Comparison of osteoclast numbers induced by individual IL-32 isoforms IL-32α, IL-32β, or IL-32γ in combination with RANKL and M-CSF, with or without TGF-β (30 ng/mL). Osteoclasts were counted using Zeiss Zen 3.2 software from 4 image clusters (3 × 3 tiles per cluster), representing 36 image tiles per condition per donor, from three independent donors. Statistical analysis was performed using nonparametric Mann–Whitney U test. Scale bars in tile images represent 100 µm in (**D**) and 50 µm in (**D**,**E**).

Images were acquired using a Zeiss AxioObserver Z1 Yokogawa CSU-X1 spinning disk confocal inverted microscope (Carl Zeiss, Jena, Germany) equipped with a motorized stage, Piezo objectives, and an Evolve EMCDD (512 × 512, 16 bit, 16 µm pixel size) monochrome camera (Photometrics). A Plan Apochromat 20×/0.8 DICII objective (Zeiss, Germany) was used to acquire the images (final pixel size of 667 nm). Excitation was processed sequentially as follows: for AF647 and AF488, 639 nm and 488 nm solid state lasers, respectively, were used, associated with a double band pass filter in emission (525/25 + LP650, from Chroma); for AF555 and DAPI excitation, 561 nm and 405 nm solid state lasers, respectively, were used, associated with a double band pass filter in emission (445/25 + 590/30 from Chroma). Four different representative regions of each coverslip were randomly acquired as a 3 × 3 (Figure 1) tiling with 10% overlap. For cell counting on tiling images acquired at 20× magnification, osteoclast cells were counted manually (Figure 1). For osteocalcin+ cell counting in Figure 2, Macro for Osteocalcin Full Analysis of Mean Fluorescence Intensity (MFI) was used. Briefly, after background subtraction (rolling = 50) and segmentation with automatic thresholding, a watershed filter was applied to separate each nucleus, and analysis particle module (size = 20–700) was used to finally count the osteoblast cells (Figure 2), whereas osteoclasts were counted manually as described for Figure 1. For osteocalcin+ cell counting in Figure 3, full wells were acquired using an EC Plan NeoFluar 10×/0.3 DICI objective under the same conditions of acquisitions as described above with the tiling module. Osteoclast cells counting in Figure 4 was carried out as in Figure 1 with 4 × 3 tiling with 10% overlap. The stitching module from Zen software (Version 3.2) was used to correct the shift in tiling images (based on Phalloidin-AF647 signal). For image analysis, macros were developed under the FIJI software with ImageJ (Fiji, ImageJ, 64-bit/https://imagej.net/software/fiji/downloads (accessed on 31 January 2025)) Macro language. All macros used are described in the Supplementary Data. All microscopy figures were generated using the QuickFigures plugin under FIJI (Fiji, ImageJ, 64-bit).

### 2.6. Quantification of Soluble Proteins

Human IL-32, TGF-β, RANKL, osteoprotegerin (OPG), and TRACP-5b were quantified from plasma of PWH and HIV-negative controls and supernatants of monocytes stimulated with recombinant IL-32 isoforms, RANKL, and/or M-CSF by ELISA as per the supplier’s instructions.

### 2.7. Statistical Analysis

Data were analyzed using GraphPad Prism 8 (GraphPad software, San Diego, CA, USA) and SAS 9.4 (SAS Institute, Cary, NC, USA). Non-parametric Kruskal–Wallis and Dunn’s subtests were used for multiple comparisons. Mann–Whitney non-parametric analysis was used to compare two groups for the same variable, whereas Wilcoxon matched-pairs signed rank test was used to compare conditions before and after treatment. Non-parametric Spearman’s test was used for correlations. Differences between groups were considered statistically significant at values of *p* < 0.05 with two-tailed analysis.

## 3. Results

### 3.1. IL-32 Isoforms Differentially Impact Classical Monocyte Osteogenic Differentiation In Vitro

Osteoclast precursors are known to be of monocyte/macrophage lineage origin that fuse together under specific stimulation conditions to form multinucleated giant cells involved in bone resorption [38,39]. Meanwhile, osteoblasts seem to have multiple cellular origins, including bone marrow stromal cells and mesenchymal stem cells [40], with limited research on the monocyte/macrophage lineage potential to generate these cells. Given the diverse roles of the different IL-32 isoforms on monocyte/macrophage activation [25,41], we aimed here to study the impact of IL-32 isoforms on the osteogenic potential of these cells. We first studied the impact of IL-32α, IL-32β, and IL-32γ (the only commercially available IL-32 recombinant isoform proteins) on the osteogenic differentiation of primary classical monocytes (CD14++CD16−) isolated from HIV-negative control participants from the Canadian Cohort of HIV and Aging Study (CHACS) [36]. Monocytes with a purity > 98% (Figure S1) were either exposed to IL-32 isoforms (α, β, and/or γ) alone (500 ng/mL) or in combinations with the prototypic osteoclast activators [42] M-CSF (25 ng/mL) and RANKL (30 ng/mL) for 21 days. The differentiated osteoclasts were visualized by multiplex spinning-disk confocal imaging and counted on the basis of TRACP5 (tartrate-resistant acid phosphatase 5, a sensitive and specific indicator for bone resorption [43]) expression (Green) and the presence of F-Actin ring (Magenta) combined with a phenotype of multiple nuclei ≥ 3 (Blue) (Figure 1). As expected, our immunostaining showed distinct osteoclast differentiation following the treatment of the monocytes (from n = 6 donors) with the osteoclast prototypic stimulators M-CSF and RANKL [44,45,46] (Figure 1A,D right panel). Similarly, cells stimulated with IL-32α alone showed a phenotype of differentiated osteoclasts (Figure 1B,D left panels, *p* < 0.0001) and their numbers increased under the combination with M-CSF + RANKL (Figure 1C, left panel and Figure 1D right panel). In contrast, the stimulation of monocytes with IL-32β or IL-32γ did not induce osteoclast differentiation, either when used alone or in combination with IL-32α or M-CSF and RANKL (Figure 1B,C, middle and right panels, respectively). The lack of osteoclast differentiation in the presence of the IL-32 inflammatory isoforms even when the strong osteogenic stimulators M-CSF and RANKL were used (Figure 1D) suggests a specific inhibitory mechanism. Indeed, immunofluorescence staining of cells stimulated with IL-32β or IL-32γ revealed that they were TRACP5-negative, mononucleated cells with an elongated cuboidal shape (Figure 1B,C, middle and right panels, respectively). In addition, the number of cells with this non-osteoclastic phenotype increased in conditions with M-CSF + RANKL (Figure 1C, middle panels), likely due to better cell survival mediated by M-CSF co-stimulation [47]. These results show that IL-32α, an isoform that we recently identified to be upregulated in women with HIV and subclinical atherosclerosis [26], favors, alone or in synergy with M-CSF and RANKL, osteoclast differentiation. Meanwhile, the dominantly expressed IL-32β and IL-32γ isoforms [25] significantly inhibit this phenomenon and instead favor a TRACP5-negative mononucleated cell phenotype, by a so-far unidentified mechanism that needs to be further characterized.

### 3.2. IL-32β and IL-32γ Induce Osteocalcin^+^ Osteoblast-like Cells

To better define how single IL-32β and L-32γ isoforms impact monocyte differentiation, we measured the osteoblast marker osteocalcin (a protein specifically produced by osteoblasts, which is abundantly found in bone tissues and reflects the osteoblast maturation and mineralizing phenotype [48,49]) via immunofluorescence staining. Primary classical monocytes (from n = 4 HIV-negative donors) were exposed to the IL-32 isoforms and osteogenic stimulations, similar to the conditions and incubation times described in Figure 1. Our data shown in Figure 2A demonstrated that cells differentiated in response to IL-32β and L-32γ acquired the expression of osteocalcin in an osteoblast-like phenotype. The morphology and osteocalcin expression by these cells matched those of matured osteocalcin+ osteoblasts isolated from human femoral heads as shown by other groups [50]. By applying automated counting using FIJI ImageJ software (Fiji, ImageJ, 64-bit) on the basis of the mononucleated phenotype combined with osteocalcin expression, our data showed that osteocalcin+ cells were significantly absent from IL-32α, M-CSF, and RANKL conditions, suggesting an exclusive expression of osteocalcin under IL-32β and IL-32γ stimulations (Figure 2A,B, *p* < 0.0001 for both). Consistent with the data shown in Figure 1, cells stimulated with IL-32α, M-CSF, and RANKL differentiated into osteoclasts with the triple phenotype (multinuclei, F-actin, and TRACP expression) (Figure 2A).

To further confirm the potential of IL-32β and IL-32γ to induce the osteoblast differentiation phenotype, we used human mesenchymal stem cell/hMSC progenitor cells isolated from human bone marrow, as described in the Materials and Methods section. These cells are multipotent, having the potential to differentiate into a number of cell types, including osteoblasts, when incubated with the appropriate cytokine combination [51]. The differentiation of hMSCs was carried out as outlined in Figure S2. As anticipated, incubating these cells with the osteoblast differentiation medium (OBDM) allowed the upregulation of osteocalcin expression following the incubation period of 21 days. No TRACP5 expression or multinuclear phenotype was observed in the differentiated cells (Figure 3A). To measure the magnitude of this effect, we acquired images of the entire culture-well for each condition at 10×, and the total numbers of osteocalcin+ cells were calculated by automated cell counting using FIJI software (Fiji, ImageJ, 64-bit) as described in the Materials and Methods section (Figure 3B, upper and lower left panels). Interestingly, treatment with either IL-32β or L-32γ, but not IL-32α, significantly increased the number of osteocalcin+ cells compared to the control conditions, where hMSCs were maintained in mesenchymal stem cell growth medium (n = 5 independent experiments) (Figure 3A,B, *p* = 0.0.0079 for both). By normalizing the number of osteocalcin+ cells from IL-32-stimulated conditions to the negative controls (the cells maintained in the growth medium only) and comparing the potential of the individual isoforms, we observed that IL-32β was the strongest isoform in the induction of osteocalcin expression (Figure S3). These observations further confirmed the potential of the inflammatory IL-32β and L-32γ isoforms in the induction of osteoblast cell differentiation.

### 3.3. IL-32β and IL-32γ Specifically Block Osteoclast Differentiation Through Inhibition of TGF-β and TRACP5b from Classical Monocytes In Vitro

Our data described above suggest that IL-32β and L-32γ isoforms may likely interfere with the formation of osteoclasts to favor the differentiation of the calcium depositing cells, osteoblasts. A crosstalk between these cell types is known to govern the fine balance between calcium deposition and resorption. This crosstalk involves multiple cytokines and ligands that include RANKL (a ligand initiating osteoclast differentiation) and its decoy receptor osteoprotegerin/OPG (a member of the TNF superfamily), which neutralizes RANKL functions [17], as well as TGF-β, which plays a critical role in osteoclast formation [52], and TRACP5b, an indicator of osteoclast function [43]. To determine whether IL-32 isoforms interfere with this crosstalk, we measured the abovementioned cytokines and ligands in supernatants from monocytes cultured under the same conditions as in Figure 1. Our results from the experiments repeated on classical monocytes isolated from n = 6 HIV-negative donors showed that IL-32α, IL-32β, and L-32γ differentially induce the production of these proteins. Cells stimulated with IL-32α produced significantly higher levels of the osteoclast-specific proteins RANKL and TRACP5b compared to cells stimulated with IL-32β and L-32γ or even compared to conditions combining the three IL-32 isoforms (Figure 4A, left and right panels, respectively). In line with these results, IL-32β and L-32γ inhibited TRACP5b production when the strong osteoclastogenic stimulators M-CSF and RANKL were used (Figure S4A). No significant difference was observed in the expression of RANKL’s decoy receptor OPG between the three isoforms (Figure 4B). However, the combination of IL-32β and L-32γ significantly decreased OPG expression in cells stimulated with M-CSF and RANKL (Figure S4B). Notably, the expression of OPG by osteoclasts themselves was previously shown to be autoregulatory and is associated with enhanced osteoclast resorbing activity [53]. Furthermore, IL-32α, in contrast to IL-32β and IL-32γ, proved to be a strong inducer of TGF-β, while IL-32β and L-32γ dramatically diminished the expression of TGF-β (Figure 4C, *p* = 0.0034 and *p* = 0.0005, respectively). Similar results were observed in cells stimulated with the IL-32β and L-32γ isoforms under the condition of osteoclast cell differentiation (cell stimulation with recombinant M-CSF and RANKL (Figure S4C)). Together, these results suggest that TGF-β might be necessary for the differentiation of osteoclasts in the current experimental conditions. Indeed, TGF-β was recently shown to promote osteoclastogenesis through a non-canonical pathway in cells primed with TNF-α [54], a proinflammatory cytokine that we showed to be highly produced by monocytes/macrophages in response to IL-32β and L-32γ, but not IL-32α [25]. To test this hypothesis, we cultured the classical monocytes (from n = 3 independent donors) in osteoclastogenic conditions, as described in Figure 1, under co-stimulation with IL-32β and IL-32γ and exogenous supplementation with recombinant TGF-β (30 ng/mL). Our data showed that TGF-β significantly reversed the dominant osteoblastogenic potential of IL-32β- and IL-32γ-stimulated cells and formed osteoclasts with the typical differentiation markers, the multinuclear, F-actin ring and TRACP expression (*p* < 0.0001 for both) (Figure 4D, lower middle and right panels; and Figure 4F, middle and right panels, respectively). As expected, cells stimulated with IL-32β and IL-32γ without TGF-β differentiated into the typical mononucleated osteocalcin+ osteoblasts (Figure 4D, upper middle and right panels; and Figure 4E, left and right panels, respectively), whereas cells stimulated with IL-32α in combination with M-CSF and RANKL differentiated into the typical osteoclasts in the presence or absence of TGF-β (Figure 4D,F, left panels). These data suggest a complex interplay between the proinflammatory isoforms of IL-32 (IL-32β and IL-32γ) and the anti-inflammatory isoform (IL-32α) as well as TGF-β in determining the fate of monocytes/macrophages to differentiate into either the calcium depositing or resorbing cells.

### 3.4. Plasma Levels of the Osteoblast/Osteoclast Regulator OPG Are Associated with Vascular Calcification and Stenosis in PWH

**Figure 5:**
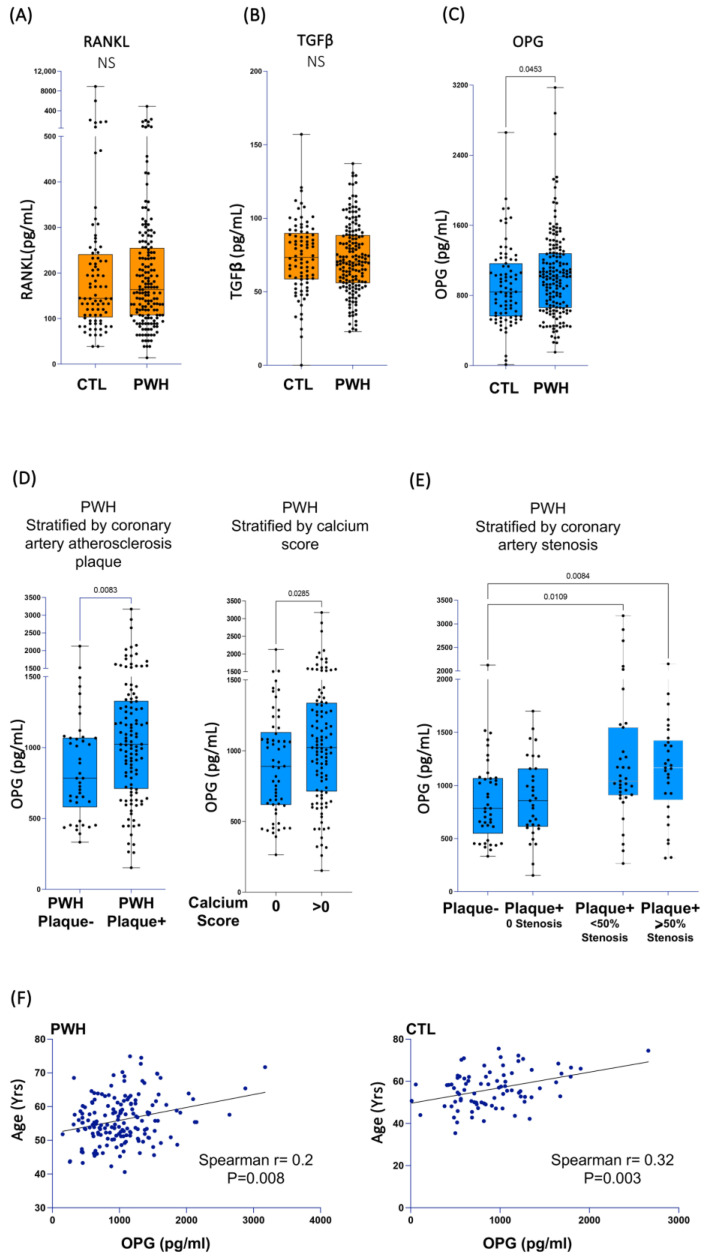
Plasma biomarkers associated with vascular calcification and subclinical atherosclerosis in PWH. Plasma collected from PWH (n = 168) and a control group (n = 84) were used to measure soluble RANKL (**A**), TGFβ (**B**), and OPG (**C**). (**D**) Left panel: OPG plasma levels in plasma from PWH stratified by presence (n = 114) or absence (n = 43) of measurable subclinical atherosclerotic plaque in coronary artery. Right panel: OPG plasma levels in plasma from PWH stratified by calcium score (data available for n = 166/n = 107 with positive calcium score and n = 59 with score = 0). (**E**) OPG plasma levels in plasma from PWH participants for whom coronary artery maximum stenosis was calculated (data available for n = 139/n = 42 with no coronary artery plaque, n = 33 with measurable plaque but zero % stenosis, n = 35 with measurable plaque and stenosis levels < 50%, and n = 29 with measurable plaque and stenosis levels ≥ 50%). (**F**) Correlation between plasma OPG levels and age of PWH (n = 168, left panel) and control group participants (n = 84, right panel). Data were analyzed using non-parametric Mann–Whitney test in (**A**–**D**), Kruskal–Wallis and Dunn’s subtest in (**E**) and Spearman’s correlations in (**F**).

Our earlier results regarding the ART-treated PWH participating in the Canadian Cohort of HIV and Aging Study (CHACS) demonstrated that the expression of all IL-32 isoforms was significantly upregulated compared to the controls [25,55]. In addition, we also showed that IL-32 expression is positively correlated with the presence of subclinical atherosclerosis [25] and with arterial stiffness [27]. Given the link between arterial stiffness and vascular calcification [28], and the role of the dominant IL-32 isoforms’ function in the differentiation of the calcifying osteoblasts, we aimed to investigate the expression of markers of vascular calcification in our cohort of PWH. To this end, we quantified the osteogenic proteins RANKL, OPG, and TGF-β in plasma collected from the CHACS participants. Table 1 summarizes the demographics and clinical characteristics of the study participants, including PWH and the control group. As depicted in Figure 5A,B, no significant difference in plasmatic levels of RANKL or TGF-β was observed between the two groups. In contrast, OPG levels were higher in PWH (Figure 5C, *p* = 0.045). We further stratified the population of PWH based on the presence or absence of coronary artery atherosclerotic plaques (the total of calcified and noncalcified plaques). As shown in Table 2, PWH in the CHACS cohort from both groups (plaque+ and plaque−) were selected to have similar cardiovascular disease risk factors, including the Framingham risk score, LDL-C and HDL-C, hsCRP, D-dimer levels, and viral load and duration of treatment. This design allowed us to investigate disease biomarkers beyond the traditional and commonly used risk factors. However, the participants with coronary artery plaque were slightly older (57 ± 6.82 years) compared to the PWH without plaque (54.5 ± 6.41 years). Under this stratification, the plasma OPG was significantly higher in the participants with plaque (Figure 5D, left panel, *p* = 0.008). Similar results were also obtained when the PWH were stratified by the presence or absence of coronary artery calcium (calcium score 0 vs. >0) (Figure 5D, right panel, *p* = 0.0285). Further stratification of the PWH based on the presence of coronary artery stenosis demonstrated that the participants with detectable stenosis (>0) had significantly higher levels of OPG (Figure 5E, *p* = 0.01 for participants with <50% stenosis and *p* = 0.008 for participants with ≥50% stenosis). Neither of these associations was observed in the control population that was also selected based on similar cardiovascular disease risk scores (Table 2 and Figure S5A,B). Intriguingly, OPG did not correlate with either IL-32 total protein measured in plasma from the study participants (both in PWH, n = 168, and the control group, n = 84) or with total IL-32 cell-associated RNA (according to the available data from our earlier studies [25] on the same participants; i.e., PWH, n = 65, and the control group, n = 44) (Figure S5C,D). Since arterial stiffness and vascular atherosclerosis increase with aging, we tested the association between OPG and age. The data shown in Figure 5F show that OPG levels are positively and significantly correlated with age, in both the PWH and control groups (*p* = 0.008 and *p* = 0.003, respectively). Together, these data suggest that OPG, a marker with mounting evidence supporting its predictive value in vascular calcification [56], is upregulated in PWH from the CHACS in whom we have recently shown that IL-32 is upregulated and is associated with vascular stiffness and CVD [25,27].

## 4. Discussion

In this study, we investigated the role of IL-32, a proinflammatory cytokine we recently showed to be associated with arterial stiffness and CVD in PWH [25,26,27], in vascular pathology, by focusing on the role of IL-32 isoforms in the activation and differentiation of osteoblasts and osteoclasts. Under physiological conditions, the co-existence and balance between these two cell types is tightly regulated to maintain bone homeostasis and to avoid net changes in bone mass [57]. Beyond the implication of these cells in bone remodeling, mounting evidence points to the implication of osteoblasts and osteoclasts in the pathophysiology of cardiovascular disease, as vascular calcification is considered to be of cellular origin [58]. Multiple inflammatory and anti-inflammatory cytokines such as TNF-α, IL-1β, IFNγ, IL-17, IL-3, and IL-10 have been shown to regulate the differentiation and/or function of osteoblasts and osteoclasts [59]. Interestingly, data generated by our group suggest that IL-32 may act upstream of these cytokines by regulating their expression. For instance, our recent data showed that the dominantly expressed IL-32 isoforms β and γ induce the expression of TNF-α, IL-6, IL-1β, IL-10, and IL-18 in monocytes/macrophages and IL-6 and IFNγ in T cells [25,55]. In contrast, the less expressed IL-32α isoform is associated with the expression of the anti-inflammatory cytokine IL-10 [55]. This differential impact on the expression of inflammatory and anti-inflammatory cytokines is also reflected in the potential of IL-32 isoforms to regulate the differentiation of monocytes into type 1 or type 2 macrophages, as IL-32α induces M2 macrophages, whereas IL-32β and γ induce M1 inflammatory macrophages upon short-term stimulation [25]. The differentiation of M2 macrophages by IL-32α is likely mediated by TGF-β, an anti-inflammatory cytokine known for its role in promoting M2 macrophage polarization [60] and that we showed here to be induced by IL-32α. Indeed, M2 macrophages were shown to fuse together under certain stimulation conditions such as with IL-4 to form osteoclasts [39,61], a phenotype that we showed in the current study under monocyte stimulation with IL-32α. This observation is consistent with earlier reports showing the potential of IL-32α to induce osteoclast differentiation from fibroblast-like synoviocytes in synergy with IL-17 [34]. However, in our work, we show that long-term stimulation with IL-32α alone with no other co-stimulators has the potential to differentiate human monocytes/macrophages into osteoclasts expressing the functional osteoclast marker TRACP. This observation is of particular interest, as it supports the role of IL-32α as a substitute for the canonical pathway involving the receptor/ligand RANK/RANKL in osteoclast differentiation [62]. Meanwhile, the function of IL-32α seems to be regulated by IL-32β and γ, since the combination of IL-32α with its counter isoforms abrogates the osteoclastogenic potential, even under conditions when the master osteoclastogenic stimulators M-CSF and RANKL [63] were used.

The reason behind the autoregulation of IL-32 functions by its own isoforms remains largely unknown. IL-32 is expressed in multiple isoforms, including IL-32α, IL-32β, IL-32γ, IL-32δ, IL-32D, IL-32ε, IL-32ξ, IL-32η, IL-32θ, and IL-32ζ, with IL-32γ being the most active and proinflammatory isoform [55,64]. The splicing of IL-32γ to shorter isoforms such as IL-32β was initially suggested to act as a switch mechanism aiming to protect against exaggerated inflammation [65]. However, our recent data demonstrated that IL-32β and IL-32γ share most of the inflammatory characteristics by inducing similar inflammatory profiles on different cell types, including CD4 T-cells, monocytes/macrophages, and endothelial cells [24,25,27,55]. This is important, particularly in the context of cardiovascular disease, as other groups showed that IL-32β and IL-32γ are dominantly expressed in atherosclerotic tissues compared to other IL-32 isoforms [66]. Therefore, in HIV infection, where all IL-32 isoforms are chronically upregulated [55,67,68], the function of IL-32β and IL-32γ in osteoblast differentiation combined with the potential role of these cells in vascular disease would correlate with the observed higher arterial stiffness and CVD burden observed in PWH [69,70,71].

In the current work, we also showed that IL-32β and IL-32γ diminish the osteoclastogenic effect of IL-32α through inhibition of TGF-β expression and that exogenous supplementation of TGF-β reverses this function. However, the role of TGF-β, a ubiquitous growth factor, in osteoblasts/osteoclasts is complex and important in retaining the dynamic balance between these cell types and seems to be contextual and depends on the cell origin and environment [72]. For instance, TGF-β may contribute to bone formation by inducing osteoprogenitor proliferation [73]. Meanwhile, in monocytes, the addition of TGF-β at the early stage of activation induces the differentiation of these cells into osteoclasts through p38 MAPK-dependent mechanisms [74]. In addition, in the presence of TNF-α, TGF-β was recently shown to bypass the canonical RANK/RANKL pathway and reprogram macrophages to differentiate into inflammatory osteoclasts [54]. This last report aligns with and supports our observations on the role of TGF-β in reversing IL-32β and IL-32γ osteoblastogenic effects, as these IL-32 isoforms are potent inducers of TNF-α by monocytes/macrophages, as we and others have previously shown [25,41,75]. Even with this interesting effect of the IL-32β and γ/TNF-α/TGF-β axis in monocyte-to-osteoclast differentiation, it is still intriguing how monocytes may differentiate into osteoblasts in response to IL-32β and γ. Indeed, the myeloid origin of calcifying cells in atherosclerosis has gained significant interest over the past two decades. For instance, circulating myeloid precursors with calcifying potential were shown to be of monocyte/macrophage lineage origin and to express CD14, CD68, and CD45 in addition to the calcifying osteoblast transcription factor Runx2 [76]. These cells were found to be associated with vascular calcification in patients with type 2 diabetes [76]. Recent data also demonstrate that osteocalcin+ CD14+ monocytes isolated from blood samples collected from the coronary artery sinus of non-obstructive coronary artery disease patients correlated positively with the level of calcification and also with the average necrotic core area, which suggests a role for these cells in plaque buildup and destabilization [77]. Together, these studies support our observations on the potential of IL-32β and γ to either induce the differentiation of classical CD14+ monocytes to osteocalcin+ cells or maintain the survival of the osteocalcin+ precursors. While these might represent two independent mechanisms, our data on the induction of osteocalcin expression by IL-32β and γ in mesenchymal stem cells (cells with a commitment to differentiate into calcifying osteoblasts) support the first scenario of IL-32β and γ being mechanistically involved in the differentiation of monocytes/macrophages into calcifying cells. However, these data should be interpreted with caution, since we acknowledge several limitations in our study that warrant further investigations. First, our in vitro model tested the impact of IL-32 isoforms on primary monocytes isolated from HIV-negative participants. This was principally done to study the exact function of the individual IL-32 isoforms in a primary cellular system not imprinted with the complex inflammatory signals present in monocytes from PWH. Future studies are planned to investigate differences in the osteoclast/osteoblast differentiation potential of monocytes from PWH vs. HIV-negative participants with and without atherosclerosis. Second, we only investigated the typical phenotypes of osteoclasts and osteoblasts. However, we used markers associated with full maturation such as TRACP (for osteoclasts) and the production of osteocalcin (for osteoblasts) to ensure potential function. Future studies should test the function of the IL-32-induced osteoclasts/osteoblasts by combining more specific markers such as RUNX2, alkaline phosphatase (ALP), and type I collagen Col1a1 [78]. Third, we studied only three out of up to ten known IL-32 isoforms, since these are the only commercially available isoforms. Although we show that the functions of IL-32β and γ isoforms seem dominant compared to IL-32α in the co-stimulation assays, it remains unknown how these isoforms may behave in the presence of other isoforms, such as IL-32D, IL-32θ, or IL-32ε, that were recently shown to be associated with subclinical atherosclerosis in PWH [25,26]. However, our in vitro model on the role of IL-32 isoforms in the differentiation of osteoblasts and the potential calcification function of these cells is supported by our observations on the presence of high plasma levels of osteoprotegerin/OPG in our cohort of PWH with subclinical atherosclerosis. These results align with data from other groups showing higher plasma levels of OPG in virally suppressed PWH on ART [12]. Interestingly, OPG was shown by multiple clinical studies to be a marker of coronary artery disease, vascular calcification, the severity of stenosis, and even the risk of cardiovascular mortality [79,80]. OPG functions as a decoy receptor for RANKL and therefore blocks the canonical osteoclast differentiation pathway [81]. Given this function, we expected that IL-32β and γ may induce OPG to inhibit osteoclast differentiation. However, our in vitro studies showed that OPG was produced by all cells activated with the different IL-32 isoforms (both osteoclasts and osteoblasts) and that OPG levels were even higher under conditions of co-stimulation with the osteoclastogenic M-CSF and RANKL. This might be explained by the fact that OPG is produced by osteoclasts themselves at the late stage of differentiation as an autoregulatory mechanism involved in function and apoptosis [53]. We also did not observe any association between plasma OPG and either the IL-32 protein or RNA levels in our cohort. This might be due to the production of OPG by multiple vascular cells such as endothelial cells and smooth muscle cells in response to vascular calcification [82]. Therefore, higher levels of IL-32β and γ expression may contribute to vascular calcification, and this in turn may induce OPG independently of the direct impact of IL-32 on the OPG expression mechanisms. Other possibilities could also involve the production of OPG in response to other inflammatory cytokines such as TNF-α and IL-1α [83,84] that are also known to be upregulated in HIV infection [85,86].

The upregulation of both IL-32 and OPG expression and their association with vascular calcification in PWH raises a paradigm as PWH are known to have a higher risk of non-calcified atherosclerotic plaque (a risk marker of plaque rupture [37,69]) as well as low bone mineral density and bone pathologies such as osteoporosis [87,88,89], which engages the opposite mechanism of decalcification. However, clinical evidence points to the co-existence and reciprocal regulation of these two opposite diseases, both in PWH and the general population [90]. For instance, higher levels of extra-coronary calcification, particularly in the mitral annular and aortic valve, were observed in men with HIV from the Multicenter AIDS Cohort Study (MACS) [91]. This higher calcification was paradoxically associated with non-calcified atherosclerotic plaque [91]. Similarly, in individuals with pre-dialysis chronic kidney disease, low bone mineral density is typically associated with the increased progression of coronary artery calcification [92]. While it remains not fully understood, some of the possible mechanisms that may explain the co-existence of these divergent pathologies could likely be related to the differential cellular composition between vascular and bone tissues. In bone, where pre-osteoclasts are abundant, RANKL stimulates bone resorption. Meanwhile, in the vasculature, pre-osteoclasts are rare, and RANKL can instead stimulate vascular myofibroblasts and smooth muscle cells to transform into an osteoblastic phenotype [93]. Additionally, vascular calcification and stiffness may decrease peripheral blood perfusion, which would then impact critical cellular recruitment needed for osteogenesis [94]. Other mechanisms may also involve the behavior of the inflammatory cytokines in the distinct inflammatory microenvironment of vascular and bone tissues. For instance, in rheumatoid arthritis, where TNF-α is predominant, the γ isoform of our cytokine of interest, IL-32, may induce osteoclast differentiation through RANKL induction from synoviocytes, leading to bone destruction. However, in ankylosis spondylitis (a condition of abnormal bone buildup), the dominant expression of IL-32γ induces osteoblast formation and bone buildup [31]. Indeed, our current data show that the presence of TGF-β significantly reverses the osteoblastic potential of IL-32γ to promote osteoclastogenesis. This might align with the potential role of osteoclasts in counterbalancing the effect of osteoblasts. This hypothesis is supported by data from other groups showing that osteoclasts are present at low numbers in the heavily calcified carotid artery atherosclerotic lesions in participants from the general population [95]. Meanwhile, in PWH, where coronary artery and carotid artery non-calcified atherosclerotic lesions are more prevalent [37,69], heightened expression of IL-32 inflammatory isoforms together with TGF-β in the lesions may play a role in excessive osteoclast formation and hence the higher burden of non-calcified plaque. This remains an open question and requires full characterization of the inflammatory/anti-inflammatory milieu in the atherosclerotic lesions in PWH.

## 5. Conclusions

In summary, our data highlight a key role for the dominantly expressed IL-32 isoforms β and γ in vascular calcification and atherosclerosis in ART-treated PWH through the differentiation of monocytes/macrophages into osteoblasts. These novel functions add to the multiple pathological mechanisms associated with the upregulation of IL-32 isoforms in PWH and support the potential consideration of this inflammatory cytokine as a therapeutic target.

## Figures and Tables

**Table 1:** Demographic and clinical parameters of study participants (PWH vs. controls).

Variable	PWH N = 168 (Female/Male) (n = 16/n = 152)	Controls N = 84 (Female/Male) (n = 23/n = 61)	*p* Value
Age (Years)	56 ± 6.8	56.4 ± 8.7	NS
Predicted 10 years Framingham Risk score	10.5 ± 5.7	10.4 ± 4.6	NS
Duration of ART (Years)	13.9 ± 6.9	N/A	
Log_10_ VL (Copies/mL)	1.7 ± 0.5	N/A	
Nadir CD4 count (Cells/mm^3^)	217 ± 153	N/A	
CD4 count (cells/mm^3^)	581 ± 255	NA	
CD4/CD8 ratio	0.85 ± 0.46	NA	
D-dimer (ug/mL)	338 ± 222.5	403 ± 288.4	NS
hsCRP (mg/L)	6.4 ± 5.1	6.4 ± 5.29	NS
LDL–C (mmol/L)	2.8 ± 0.9	3.3 ± 0.9	0.0005
HDL–C (mmol/L)	1.3 ± 0.3	1.4 ± 0.3	0.0003

Numbers are shown as mean ± SD. N/A: non-applicable. NA: non-available. NS: non-significant.

**Table 2:** Demographic and clinical parameters of study participants stratified by presence or absence of subclinical atherosclerosis.

Variable	PWH Plaque− N = 43 (Female/Male) (n = 4/n = 39)	PWH Plaque+ N = 114 (Female/Male) (n = 10/n = 104)	*p* Value	Controls Plaque− N = 35 (Female/Male) (n = 9/n = 28)	Controls Plaque+ N = 47 (Female/Male) (n = 14/n = 33)	*p* Value
Age (Years)	54.5 ± 6.41	57 ± 6.82	0.07	53.32 ± 7.11	58.7 ± 9.26	0.01
Predicted 10 years Framingham Risk score	9.6 ± 4.95	10.9 ± 6.16	NS	8.7 ± 4.25	11.6 ± 4.58	0.006
Statin use	2	20		1	1	
History of clinical CVD	2	8		0	0	
Duration of ART (Years)	13 ± 7.1	14.3 ± 6.79	NS	N/A	N/A	
Log_10_ VL (Copies/mL)	1.6 ± 0.45	1.63 ± 0.25	NS	N/A	N/A	
Nadir CD4 count (Cells/mm^3^)	216 ± 165	219 ± 152	NS	N/A	N/A	
CD4 count (cells/mm^3^)	606 ± 257	575 ± 242	NS	NA	NA	
CD4/CD8 ratio	0.9 ± 0.49	0.8 ± 0.45	NS	NA	NA	
D-dimer (ug/mL)	246.8 ± 77.1	339.5 ± 235.08	NS	470 ± 459.5	373.5 ± 186.14	NS
hsCRP (mg/L)	6.2 ± 3.8	5.9 ± 3.83	NS	6.6 ± 6.54	6.29 ± 4.65	
LDL–C (mmol/L)	2.9 ± 0.73	2.8 ± 0.95	NS	3.25 ± 0.86	3.3 ± 0.99	NS
HDL–C (mmol/L)	1.3 ± 0.4	1.3 ± 0.31	NS	1.4 ± 0.41	1.4 ± 0.32	NS

Numbers are shown as mean ± SD. N/A: non-applicable. NA: non-available. NS: non-significant.

## Data Availability

Original data generated in this current work can be accessed through Mendeley data repository with DOI number 10.17632/f97gr7w6ck.1 (public access date: 18 September 2025).

## Supplementary Materials

The following supporting information can be downloaded at: https://www.mdpi.com/article/10.3390/cells14070481/s1, Table S1: References for key resources; Figure S1: Flowcytometry Gating strategy on classical monocytes (CD14++CD16−); Figure S2: A diagram showing the differentiation process of human mesenchymal stem cells/hMSC in the presence or absence of IL-32 isoforms. Figure S3: Comparison between the impact of individual IL-32 isoforms IL-32α, IL-32β, and IL-32γ on the differentiation of human mesenchymal stem cells (hMSCs) into osteoblasts. Figure S4: Impact of IL-32 isoforms on the expression of soluble osteoclast activation proteins; Figure S5: Plasma levels of OPG in control participants and correlations with IL-32.

## Abbreviations

The following abbreviations are used in this manuscript:

RANKL	receptor activator of nuclear factor kappa beta (NFkB) ligand
M-CSF	macrophage colony-stimulating factor
TRAP	tartrate-resistant acid phosphatase
hMSC	human mesenchymal stem cells
MSCGM	mesenchymal stem cell growth medium
OBDM	osteoblast differentiation medium
OPG	osteoprotegerin
CT scan	computed tomography scan

## References

[1] Aberg J.A. (2012). Aging, inflammation, and HIV infection. Top. Antivir. Med..

[2] Hunt P.W. (2014). HIV and aging: Emerging research issues. Curr. Opin. HIV AIDS.

[3] Ruzicka D.J., Imai K., Takahashi K., Naito T. (2018). Comorbidities and the use of comedications in people living with HIV on antiretroviral therapy in Japan: A cross-sectional study using a hospital claims database. BMJ Open.

[4] Zicari S., Sessa L., Cotugno N., Ruggiero A., Morrocchi E., Concato C., Rocca S., Zangari P., Manno E.C., Palma P. (2019). Immune Activation, Inflammation, and Non-AIDS Co-Morbidities in HIV-Infected Patients under Long-Term ART. Viruses.

[5] Guzman-Fulgencio M., Medrano J., Rallon N., Echeverria-Urabayen A., Miguel Benito J., Restrepo C., Garcia-Alvarez M., Vispo E., San Roman J., Sanchez-Piedra C. (2011). Soluble markers of inflammation are associated with Framingham scores in HIV-infected patients on suppressive antiretroviral therapy. J. Infect..

[6] Deeks S.G., Lewin S.R., Havlir D.V. (2013). The end of AIDS: HIV infection as a chronic disease. Lancet.

[7] Szmitko P.E., Wang C.H., Weisel R.D., de Almeida J.R., Anderson T.J., Verma S. (2003). New markers of inflammation and endothelial cell activation: Part I. Circulation.

[8] Szmitko P.E., Wang C.H., Weisel R.D., Jeffries G.A., Anderson T.J., Verma S. (2003). Biomarkers of vascular disease linking inflammation to endothelial activation: Part II. Circulation.

[9] Tabas I., Lichtman A.H. (2017). Monocyte-Macrophages and T Cells in Atherosclerosis. Immunity.

[10] Erlandson K.M., O’Riordan M., Labbato D., McComsey G.A. (2014). Relationships between inflammation, immune activation, and bone health among HIV-infected adults on stable antiretroviral therapy. J. Acquir. Immune Defic. Syndr..

[11] Merlini E., Luzi K., Suardi E., Barassi A., Cerrone M., Martinez J.S., Bai F., D’Eril G.V., Monforte A.D., Marchetti G. (2012). T-cell phenotypes, apoptosis and inflammation in HIV+ patients on virologically effective cART with early atherosclerosis. PLoS ONE.

[12] D’Abramo A., Zingaropoli M.A., Oliva A., D’Agostino C., Al Moghazi S., De Luca G., Iannetta M., d’Ettorre G., Ciardi M.R., Mastroianni C.M. (2016). Higher Levels of Osteoprotegerin and Immune Activation/Immunosenescence Markers Are Correlated with Concomitant Bone and Endovascular Damage in HIV-Suppressed Patients. PLoS ONE.

[13] Sprini D., Rini G.B., Di Stefano L., Cianferotti L., Napoli N. (2014). Correlation between osteoporosis and cardiovascular disease. Clin. Cases Miner. Bone Metab..

[14] Anagnostis P., Karagiannis A., Kakafika A.I., Tziomalos K., Athyros V.G., Mikhailidis D.P. (2009). Atherosclerosis and osteoporosis: Age-dependent degenerative processes or related entities?. Osteoporos. Int..

[15] Freedman B.I., Bowden D.W., Ziegler J.T., Langefeld C.D., Lehtinen A.B., Rudock M.E., Lenchik L., Hruska K.A., Register T.C., Carr J.J. (2009). Bone morphogenetic protein 7 (BMP7) gene polymorphisms are associated with inverse relationships between vascular calcification and BMD: The Diabetes Heart Study. J. Bone Miner. Res..

[16] Rubin M.R., Silverberg S.J. (2004). Vascular calcification and osteoporosis—The nature of the nexus. J. Clin. Endocrinol. Metab..

[17] Kim J.M., Lin C., Stavre Z., Greenblatt M.B., Shim J.H. (2020). Osteoblast-Osteoclast Communication and Bone Homeostasis. Cells.

[18] Weaver J. (2013). Insights into how calcium forms plaques in arteries pave the way for new treatments for heart disease. PLoS Biol..

[19] Boyce B.F., Li P., Yao Z., Zhang Q., Badell I.R., Schwarz E.M., O’Keefe R.J., Xing L. (2005). TNF-alpha and pathologic bone resorption. Keio J. Med..

[20] Kotake S., Sato K., Kim K.J., Takahashi N., Udagawa N., Nakamura I., Yamaguchi A., Kishimoto T., Suda T., Kashiwazaki S. (1996). Interleukin-6 and soluble interleukin-6 receptors in the synovial fluids from rheumatoid arthritis patients are responsible for osteoclast-like cell formation. J. Bone Miner. Res..

[21] Pfeilschifter J., Chenu C., Bird A., Mundy G.R., Roodman G.D. (1989). Interleukin-1 and tumor necrosis factor stimulate the formation of human osteoclastlike cells in vitro. J. Bone Miner. Res..

[22] Biasillo G., Leo M., Della Bona R., Biasucci L.M. (2010). Inflammatory biomarkers and coronary heart disease: From bench to bedside and back. Intern. Emerg. Med..

[23] De Pablo-Bernal R.S., Ruiz-Mateos E., Rosado I., Dominguez-Molina B., Alvarez-Rios A.I., Carrillo-Vico A., De La Rosa R., Delgado J., Munoz-Fernandez M.A., Leal M. (2014). TNF-alpha levels in HIV-infected patients after long-term suppressive cART persist as high as in elderly, HIV-uninfected subjects. J. Antimicrob. Chemother..

[24] El-Far M., Kouassi P., Sylla M., Zhang Y., Fouda A., Fabre T., Goulet J.P., van Grevenynghe J., Lee T., Singer J. (2016). Proinflammatory isoforms of IL-32 as novel and robust biomarkers for control failure in HIV-infected slow progressors. Sci. Rep..

[25] El-Far M., Durand M., Turcotte I., Larouche-Anctil E., Sylla M., Zaidan S., Chartrand-Lefebvre C., Bunet R., Ramani H., Sadouni M. (2021). Upregulated IL-32 Expression And Reduced Gut Short Chain Fatty Acid Caproic Acid in People Living With HIV With Subclinical Atherosclerosis. Front. Immunol..

[26] El-Far M., Hanna D.B., Durand M., Larouche-Anctil E., Sylla M., Chartrand-Lefebvre C., Cloutier G., Goulet J.P., Kassaye S., Karim R. (2021). Brief Report: Subclinical Carotid Artery Atherosclerosis Is Associated with Increased Expression of Peripheral Blood IL-32 Isoforms Among Women Living with HIV. J. Acquir. Immune Defic. Syndr..

[27] Bunet R., Roy-Cardinal M.H., Ramani H., Cleret-Buhot A., Durand M., Chartrand-Lefebvre C., Routy J.P., Thomas R., Trottier B., Ancuta P. (2023). Differential Impact of IL-32 Isoforms on the Functions of Coronary Artery Endothelial Cells: A Potential Link with Arterial Stiffness and Atherosclerosis. Viruses.

[28] Van den Bergh G., Opdebeeck B., D’Haese P.C., Verhulst A. (2019). The Vicious Cycle of Arterial Stiffness and Arterial Media Calcification. Trends Mol. Med..

[29] Bundy K., Boone J., Simpson C.L. (2021). Wnt Signaling in Vascular Calcification. Front. Cardiovasc. Med..

[30] Yao H., Sun Z., Zang G., Zhang L., Hou L., Shao C., Wang Z. (2021). Epidemiological Research Advances in Vascular Calcification in Diabetes. J. Diabetes Res..

[31] Kwon O.C., Kim S., Hong S., Lee C.K., Yoo B., Chang E.J., Kim Y.G. (2018). Role of IL-32 Gamma on Bone Metabolism in Autoimmune Arthritis. Immune Netw..

[32] Lee E.J., Lee E.J., Chung Y.H., Song D.H., Hong S., Lee C.K., Yoo B., Kim T.H., Park Y.S., Kim S.H. (2015). High level of interleukin-32 gamma in the joint of ankylosing spondylitis is associated with osteoblast differentiation. Arthritis Res. Ther..

[33] Ribeiro-Dias F., Saar Gomes R., de Lima Silva L.L., Dos Santos J.C., Joosten L.A. (2017). Interleukin 32: A novel player in the control of infectious diseases. J. Leukoc. Biol..

[34] Mabilleau G., Sabokbar A. (2009). Interleukin-32 promotes osteoclast differentiation but not osteoclast activation. PLoS ONE.

[35] Moon Y.M., Yoon B.Y., Her Y.M., Oh H.J., Lee J.S., Kim K.W., Lee S.Y., Woo Y.J., Park K.S., Park S.H. (2012). IL-32 and IL-17 interact and have the potential to aggravate osteoclastogenesis in rheumatoid arthritis. Arthritis Res. Ther..

[36] Giguere K., Chartrand-Lefebvre C., Baril J.G., Conway B., El-Far M., Falutz J., Harris M., Jenabian M.A., Leipsic J., Loutfy M. (2023). Baseline characteristics of a prospective cohort study of aging and cardiovascular diseases among people living with HIV. HIV Med..

[37] Boldeanu I., Sadouni M., Mansour S., Baril J.G., Trottier B., Soulez G., Chin A.S., Leipsic J., Tremblay C., Durand M. (2021). Prevalence and Characterization of Subclinical Coronary Atherosclerotic Plaque with CT among Individuals with HIV: Results from the Canadian HIV and Aging Cohort Study. Radiology.

[38] Fujikawa Y., Quinn J.M., Sabokbar A., McGee J.O., Athanasou N.A. (1996). The human osteoclast precursor circulates in the monocyte fraction. Endocrinology.

[39] Udagawa N., Takahashi N., Akatsu T., Tanaka H., Sasaki T., Nishihara T., Koga T., Martin T.J., Suda T. (1990). Origin of osteoclasts: Mature monocytes and macrophages are capable of differentiating into osteoclasts under a suitable microenvironment prepared by bone marrow-derived stromal cells. Proc. Natl. Acad. Sci. USA.

[40] Mizoguchi T., Ono N. (2021). The diverse origin of bone-forming osteoblasts. J. Bone Miner. Res..

[41] Shim S., Lee S., Hisham Y., Kim S., Nguyen T.T., Taitt A.S., Hwang J., Jhun H., Park H.Y., Lee Y. (2022). Comparison of the Seven Interleukin-32 Isoforms’ Biological Activities: IL-32theta Possesses the Most Dominant Biological Activity. Front. Immunol..

[42] Glantschnig H., Fisher J.E., Wesolowski G., Rodan G.A., Reszka A.A. (2003). M-CSF, TNFalpha and RANK ligand promote osteoclast survival by signaling through mTOR/S6 kinase. Cell Death Differ..

[43] Halleen J.M., Alatalo S.L., Janckila A.J., Woitge H.W., Seibel M.J., Vaananen H.K. (2001). Serum tartrate-resistant acid phosphatase 5b is a specific and sensitive marker of bone resorption. Clin. Chem..

[44] Nakashima T., Hayashi M., Takayanagi H. (2012). New insights into osteoclastogenic signaling mechanisms. Trends Endocrinol. Metab..

[45] Boyle W.J., Simonet W.S., Lacey D.L. (2003). Osteoclast differentiation and activation. Nature.

[46] Teitelbaum S.L., Ross F.P. (2003). Genetic regulation of osteoclast development and function. Nat. Rev. Genet..

[47] Wang Y., Mo X., Piper M.G., Wang H., Parinandi N.L., Guttridge D., Marsh C.B. (2011). M-CSF induces monocyte survival by activating NF-kappaB p65 phosphorylation at Ser276 via protein kinase C. PLoS ONE.

[48] Hopyan S., Gokgoz N., Bell R.S., Andrulis I.L., Alman B.A., Wunder J.S. (1999). Expression of osteocalcin and its transcriptional regulators core-binding factor alpha 1 and MSX2 in osteoid-forming tumours. J. Orthop. Res..

[49] Komori T. (2020). Functions of Osteocalcin in Bone, Pancreas, Testis, and Muscle. Int. J. Mol. Sci..

[50] Weidner H., Yuan Gao V., Dibert D., McTague S., Eskander M., Duncan R., Wang L., Nohe A. (2019). CK2.3, a Mimetic Peptide of the BMP Type I Receptor, Increases Activity in Osteoblasts over BMP2. Int. J. Mol. Sci..

[51] Phillips J.E., Petrie T.A., Creighton F.P., Garcia A.J. (2010). Human mesenchymal stem cell differentiation on self-assembled monolayers presenting different surface chemistries. Acta Biomater..

[52] Fox S.W., Haque S.J., Lovibond A.C., Chambers T.J. (2003). The possible role of TGF-beta-induced suppressors of cytokine signaling expression in osteoclast/macrophage lineage commitment in vitro. J. Immunol..

[53] Kang J.H., Ko H.M., Moon J.S., Yoo H.I., Jung J.Y., Kim M.S., Koh J.T., Kim W.J., Kim S.H. (2014). Osteoprotegerin expressed by osteoclasts: An autoregulator of osteoclastogenesis. J. Dent. Res..

[54] Xia Y., Inoue K., Du Y., Baker S.J., Reddy E.P., Greenblatt M.B., Zhao B. (2022). TGFbeta reprograms TNF stimulation of macrophages towards a non-canonical pathway driving inflammatory osteoclastogenesis. Nat. Commun..

[55] Zaidan S.M., Leyre L., Bunet R., Larouche-Anctil E., Turcotte I., Sylla M., Chamberland A., Chartrand-Lefebvre C., Ancuta P., Routy J.P. (2019). Upregulation of IL-32 Isoforms in Virologically Suppressed HIV-Infected Individuals: Potential Role in Persistent Inflammation and Transcription From Stable HIV-1 Reservoirs. J. Acquir. Immune Defic. Syndr..

[56] Van Campenhout A., Golledge J. (2009). Osteoprotegerin, vascular calcification and atherosclerosis. Atherosclerosis.

[57] Feng X., McDonald J.M. (2011). Disorders of bone remodeling. Annu. Rev. Pathol..

[58] Jiang W., Zhang Z., Li Y., Chen C., Yang H., Lin Q., Hu M., Qin X. (2021). The Cell Origin and Role of Osteoclastogenesis and Osteoblastogenesis in Vascular Calcification. Front. Cardiovasc. Med..

[59] Xu J., Yu L., Liu F., Wan L., Deng Z. (2023). The effect of cytokines on osteoblasts and osteoclasts in bone remodeling in osteoporosis: A review. Front. Immunol..

[60] Zhang F., Wang H., Wang X., Jiang G., Liu H., Zhang G., Wang H., Fang R., Bu X., Cai S. (2016). TGF-beta induces M2-like macrophage polarization via SNAIL-mediated suppression of a pro-inflammatory phenotype. Oncotarget.

[61] Nie Z., Hu Z., Guo X., Xiao Y., Liu X., de Bruijn J.D., Bao C., Yuan H. (2023). Genesis of osteoclasts on calcium phosphate ceramics and their role in material-induced bone formation. Acta Biomater..

[62] Takegahara N., Kim H., Choi Y. (2022). RANKL biology. Bone.

[63] Takayanagi H. (2021). RANKL as the master regulator of osteoclast differentiation. J. Bone Miner. Metab..

[64] Hong J.T., Son D.J., Lee C.K., Yoon D.Y., Lee D.H., Park M.H. (2017). Interleukin 32, inflammation and cancer. Pharmacol. Ther..

[65] Heinhuis B., Netea M.G., van den Berg W.B., Dinarello C.A., Joosten L.A. (2012). Interleukin-32: A predominantly intracellular proinflammatory mediator that controls cell activation and cell death. Cytokine.

[66] Heinhuis B., Popa C.D., van Tits B.L., Kim S.H., Zeeuwen P.L., van den Berg W.B., van der Meer J.W., van der Vliet J.A., Stalenhoef A.F., Dinarello C.A. (2013). Towards a role of interleukin-32 in atherosclerosis. Cytokine.

[67] Nold M.F., Nold-Petry C.A., Pott G.B., Zepp J.A., Saavedra M.T., Kim S.H., Dinarello C.A. (2008). Endogenous IL-32 controls cytokine and HIV-1 production. J. Immunol..

[68] Rasool S.T., Tang H., Wu J., Li W., Mukhtar M.M., Zhang J., Mu Y., Xing H.X., Wu J., Zhu Y. (2008). Increased level of IL-32 during human immunodeficiency virus infection suppresses HIV replication. Immunol. Lett..

[69] Roy Cardinal M.H., Durand M., Chartrand-Lefebvre C., Fortin C., Baril J.G., Trottier B., Routy J.P., Soulez G., Tremblay C., Cloutier G. (2020). Increased carotid artery wall stiffness and plaque prevalence in HIV infected patients measured with ultrasound elastography. Eur. Radiol..

[70] Lo J., Abbara S., Shturman L., Soni A., Wei J., Rocha-Filho J.A., Nasir K., Grinspoon S.K. (2010). Increased prevalence of subclinical coronary atherosclerosis detected by coronary computed tomography angiography in HIV-infected men. AIDS.

[71] Marcus J.L., Leyden W.A., Alexeeff S.E., Anderson A.N., Hechter R.C., Hu H., Lam J.O., Towner W.J., Yuan Q., Horberg M.A. (2020). Comparison of Overall and Comorbidity-Free Life Expectancy Between Insured Adults with and Without HIV Infection, 2000–2016. JAMA Netw. Open.

[72] Janssens K., ten Dijke P., Janssens S., Van Hul W. (2005). Transforming growth factor-beta1 to the bone. Endocr. Rev..

[73] Borton A.J., Frederick J.P., Datto M.B., Wang X.F., Weinstein R.S. (2001). The loss of Smad3 results in a lower rate of bone formation and osteopenia through dysregulation of osteoblast differentiation and apoptosis. J. Bone Miner. Res..

[74] Karsdal M.A., Hjorth P., Henriksen K., Kirkegaard T., Nielsen K.L., Lou H., Delaisse J.M., Foged N.T. (2003). Transforming growth factor-beta controls human osteoclastogenesis through the p38 MAPK and regulation of RANK expression. J. Biol. Chem..

[75] Fahmi H., Charon D., Mondange M., Chaby R. (1995). Endotoxin-induced desensitization of mouse macrophages is mediated in part by nitric oxide production. Infect. Immun..

[76] Fadini G.P., Albiero M., Menegazzo L., Boscaro E., Vigili de Kreutzenberg S., Agostini C., Cabrelle A., Binotto G., Rattazzi M., Bertacco E. (2011). Widespread increase in myeloid calcifying cells contributes to ectopic vascular calcification in type 2 diabetes. Circ. Res..

[77] Collin J., Gossl M., Matsuo Y., Cilluffo R.R., Flammer A.J., Loeffler D., Lennon R.J., Simari R.D., Spoon D.B., Erbel R. (2015). Osteogenic monocytes within the coronary circulation and their association with plaque vulnerability in patients with early atherosclerosis. Int. J. Cardiol..

[78] Huang W., Yang S., Shao J., Li Y.P. (2007). Signaling and transcriptional regulation in osteoblast commitment and differentiation. Front. Biosci..

[79] Jono S., Ikari Y., Shioi A., Mori K., Miki T., Hara K., Nishizawa Y. (2002). Serum osteoprotegerin levels are associated with the presence and severity of coronary artery disease. Circulation.

[80] Kiechl S., Werner P., Knoflach M., Furtner M., Willeit J., Schett G. (2006). The osteoprotegerin/RANK/RANKL system: A bone key to vascular disease. Expert Rev. Cardiovasc. Ther..

[81] Lacey D.L., Timms E., Tan H.L., Kelley M.J., Dunstan C.R., Burgess T., Elliott R., Colombero A., Elliott G., Scully S. (1998). Osteoprotegerin ligand is a cytokine that regulates osteoclast differentiation and activation. Cell.

[82] Collin-Osdoby P. (2004). Regulation of vascular calcification by osteoclast regulatory factors RANKL and osteoprotegerin. Circ. Res..

[83] Hofbauer L.C., Dunstan C.R., Spelsberg T.C., Riggs B.L., Khosla S. (1998). Osteoprotegerin production by human osteoblast lineage cells is stimulated by vitamin D, bone morphogenetic protein-2, and cytokines. Biochem. Biophys. Res. Commun..

[84] Vidal O.N., Sjogren K., Eriksson B.I., Ljunggren O., Ohlsson C. (1998). Osteoprotegerin mRNA is increased by interleukin-1 alpha in the human osteosarcoma cell line MG-63 and in human osteoblast-like cells. Biochem. Biophys. Res. Commun..

[85] Kumar A., Coquard L., Herbein G. (2016). Targeting TNF-Alpha in HIV-1 Infection. Curr. Drug Targets.

[86] Hoel H., Dahl T.B., Yang K., Skeie L.G., Michelsen A.E., Ueland T., Damas J.K., Dyrhol-Riise A.M., Fevang B., Yndestad A. (2024). Chronic HIV Infection Increases Monocyte NLRP3 Inflammasome-Dependent IL-1alpha and IL-1beta Release. Int. J. Mol. Sci..

[87] Hileman C.O., Eckard A.R., McComsey G.A. (2015). Bone loss in HIV: A contemporary review. Curr. Opin. Endocrinol. Diabetes Obes..

[88] Pramukti I., Lindayani L., Chen Y.C., Yeh C.Y., Tai T.W., Fetzer S., Ko N.Y. (2020). Bone fracture among people living with HIV: A systematic review and meta-regression of prevalence, incidence, and risk factors. PLoS ONE.

[89] Compston J. (2014). Osteoporosis and fracture risk associated with HIV infection and treatment. Endocrinol. Metab. Clin. N. Am..

[90] Cannata-Andia J.B., Roman-Garcia P., Hruska K. (2011). The connections between vascular calcification and bone health. Nephrol. Dial. Transplant..

[91] Rezaeian P., Miller P.E., Haberlen S.A., Razipour A., Bahrami H., Castillo R., Witt M.D., Kingsley L., Palella F.J., Nakanishi R. (2016). Extra-coronary calcification (aortic valve calcification, mitral annular calcification, aortic valve ring calcification and thoracic aortic calcification) in HIV seropositive and seronegative men: Multicenter AIDS Cohort Study. J. Cardiovasc. Comput. Tomogr..

[92] Kim H., Lee J., Lee K.B., Kim Y.H., Hong N., Park J.T., Han S.H., Kang S.W., Choi K.H., Oh K.H. (2022). Low bone mineral density is associated with coronary arterial calcification progression and incident cardiovascular events in patients with chronic kidney disease. Clin. Kidney J..

[93] Pawade T.A., Newby D.E., Dweck M.R. (2015). Calcification in Aortic Stenosis: The Skeleton Key. J. Am. Coll. Cardiol..

[94] Gebre A.K., Lewis J.R., Leow K., Szulc P., Scott D., Ebeling P.R., Sim M., Wong G., Lim W.H., Schousboe J.T. (2023). Abdominal Aortic Calcification, Bone Mineral Density, and Fractures: A Systematic Review and Meta-analysis of Observational Studies. J. Gerontol. A Biol. Sci. Med. Sci..

[95] Qiao J.H., Mishra V., Fishbein M.C., Sinha S.K., Rajavashisth T.B. (2015). Multinucleated giant cells in atherosclerotic plaques of human carotid arteries: Identification of osteoclast-like cells and their specific proteins in artery wall. Exp. Mol. Pathol..

