# Supplementary material for: Opposite Roles of IL-32α Versus IL-32β/γ Isoforms in Promoting Monocyte-Derived Osteoblast/Osteoclast Differentiation and Vascular Calcification in People with HIV
Source: Cells. 2025 Mar 22;14(7):481. doi: 10.3390/cells14070481 (PMC11987946; doi:10.3390/cells14070481)
Supplement: Supplementary file 1 [file cells-14-00481-s001.zip › cells-3483000-supplementary.pdf]

Opposite Roles of IL-32α Versus IL-32β/γ Isoforms in Promoting Monocyte-Derived Osteoblast/Osteoclast Differentiation and Vascular Calcification in People with HIV

Hardik Ramani [1,2], Aurélie Cleret-Buhot [2,3], Mohamed Sylla [2], Rémi Bunet [1,2], Florent Bertrand [1,2], Marc-Messier Peet [2], Carl Chartrand-Lefebvre [2,4], Benoit Trottier [5], Réjean Thomas [6], Jean-Pierre Routy [7], Claude Fortin [1], Valérie Martel-Laferrière [1,2], Manel Sadouni [2,3], Guy Cloutier [2,4], Louise Allard [2], Jorge R. Kizer [8], Nicolas Chomont [1,2], Petronela Ancuta [1,2], David B. Hanna [9], Robert C. Kaplan [9,10], Mohammad-Ali Jenabian [11], Alan L. Landay [12], Madeleine Durand [2,13], Mohamed El-Far [2,*,†] and Cécile L. Tremblay [1,2,*,†]

[1] Département de Microbiologie, Infectiologie et Immunologie, Faculté de Médecine, Université de Montréal, Montréal, QC H3C 3J7, Canada; hardik.ramani@umontreal.ca (H.R.); remi.bunet@umontreal.ca (R.B.); florent.bertrand@umontreal.ca (F.B.); claude.fortin.med@ssss.gouv.qc.ca (C.F.); valerie.martel-laferriere.med@ssss.gouv.qc.ca (V.M.-L.); nicolas.chomont@umontreal.ca (N.C.); petronela.ancuta@umontreal.ca (P.A.)

[2] Centre de Recherche du Centre Hospitalier de l'Université de Montréal (CRCHUM), Montréal, QC H2X 0A9, Canada; aurelie.cleret-buhot.chum@ssss.gouv.qc.ca (A.C.-B.); syllmoh@yahoo.fr (M.S.); marc.messier-peet.chum@ssss.gouv.qc.ca (M.-M.P.); carl.chartrand-lefebvre@umontreal.ca (C.C.-L.); sadounamine@outlook.fr (M.S.); guy.cloutier@umontreal.ca (G.C.); louise.allard.chum@ssss.gouv.qc.ca (L.A.); madeleine.durand@umontreal.ca (M.D.)

[3] Cellular Imaging Core Facility, Centre de Recherche du Centre Hospitalier de l'Université de Montréal (CRCHUM), Montréal, QC H2X 0A9, Canada

[4] Département de Radiologie, Radio-Oncologie et Médecine Nucléaire, Faculté de Médecine, Université de Montréal, Montréal, QC H3T 1J4, Canada

[5] Centre de Médecine Urbaine du Quartier Latin, Montréal, QC H2L 0B1, Canada; bentrotte@gmail.com

[6] Clinique Médicale l'Actuel, Montréal, QC H2L 4P9, Canada; rejean.thomas@lactuel.ca

[7] Research Institute of McGill University Health Centre, Montréal, QC H4A 3J1, Canada; jean-pierre.routy@mcgill.ca

[8] Cardiology Section, San Francisco Veterans Affairs Health Care System, and Department of Medicine, Epidemiology and Biostatistics, the University of California San Francisco, San Francisco, CA 94121, USA; jorge.kizer@ucsf.edu

[9] Department of Epidemiology and Population Health, Albert Einstein College of Medicine, Bronx, NY 10461, USA; david.hanna@einsteinmed.edu (D.B.H.); robert.kaplan@einsteinmed.edu (R.C.K.)

[10] Divsion of Public Health Sciences, Fred Hutchinson Cancer Research Center, Seattle, WA 98109, USA

[11] Department of Biological Sciences, Université du Québec à Montréal, Montréal, QC H2X 1Y4, Canada; jenabian.mohammad-ali@uqam.ca

[12] Department of Internal Medicine and Microbiology and Immunology, University of Texas, Medical Branch, Austin, TX 77555, USA; allanday@utmb.edu

[13] Département de Médecine, Faculté de Médecine, Université de Montréal, QC H3T 1J4, Canada

* Correspondence: mohamed.el.far.chum@ssss.gouv.qc.ca (M.E.-F.); c.tremblay@umontreal.ca (C.L.T.);
Tel.: +1-514-890-8000 (ext. 31241) (M.E.-F.); +1-514-890-8000 (C.L.T.)
† These authors contributed equally to this work.

Supplemental materials

Supplemental Table S1: References for key resources.

REAGENT or RESOURCES	SOURCE	IDENTIFIER
**Antibodies**		
Anti-human Tartrate Resistant Acid Phosphatase (TRAP/ACP5,14G6)	Kerafast	Cat# EUL002
Alexa Fluor™ 647 Phalloidin	Invitrogen™	Cat# A22287
Anti-human Osteocalcin Polyclonal Antibody	Invitrogen™	Cat# PA5-96529
Donkey anti-Mouse IgG (H+L) Highly Cross-Adsorbed Secondary Antibody, Alexa Fluor 488	Invitrogen™	Cat# A-21202
Anti-rabbit IgG (H+L), F(ab')2 Fragment, Alexa Fluor® 555 Conjugate	Cell Signaling technologies	Cat# 4413S
BV605-conjugated mouse anti-human CD3 (Clone: UCHT1)	BD Bioscience	Cat#742623
APC-conjugated mouse anti-human CD14	BD Bioscience	Cat#555399
**Biological Samples**		
Peripheral blood mononuclear cells (PBMCs) (HIVneg and HIV+ individuals)	Canadian HIV and Aging Cohort Study (CHACS)	
Plasma (HIVneg and HIV+ individuals)	Canadian HIV and Aging Cohort Study (CHACS)	
Human mesenchymal stem cells (hMSC)	LONZA	Cat# PT-2501
**Chemicals, Recombinant proteins and Media**		

PBS 1X (without calcium or magnesium)	MULTICELL	Cat# 311-010-LL
Paraformaldehyde (PFA)	Thermo Scientific	Cat# 41678-5000
Penicillin-Streptomycin (5,000 U/mL)	Gibco™	Cat# 15070063
DAPI (4',6-Diamidino-2-phenylindole dihydrochloride)	Sigma-Aldrich	Cat# D9542
FBS - (FETAL BOVINE SERUM), 500 ml	wisent bioproducts	Cat# 080-450
Sterile Water, molecular grade (500ml)	Multicell	Cat# 809-115-CL
EasySep™ Buffer Cell separation buffer	STEMCELL Technologies	Cat# 20144
Recombinant Human IL-32α	R&D Systems	Cat# 3040-IL-050
Recombinant Human IL-32β	R&D Systems	Cat# 6769-IL-025
Recombinant Human IL-32γ	R&D Systems	Cat# 4690-IL-025/CF
Recombinant human macrophage colony stimulating factor (M-CSF)	SIGMA-ALDRICH	Cat# M6518
Recombinant human sRANKL	Invitrogen™	Cat#PHP0034
Recombinant human TGF-β	R&D Systems	Cat# 11409-BH
Polymyxin-B	InvivoGen	Cat# tlrl-pmb
α-MEM medium	Gibco™	Cat# 12561049
Mesenchymal stem cell basal medium	LONZA	Cat# PT-3238
ProLong™ Gold antifade mountant	Invitrogen™	Cat# P36934
Triton-X-100	VWR life science	Cat#0694
Microscope cover Glass – circle #1.0	Fisher Scientific	Cat#12-545-80
Black clear bottom 96 well plate	Corning®	Cat# 3603
**Continue…**		
Costar® 24-well Clear TC-treated Multiple Well Plates, Sterile	Costar Corning	Cat# 3526

REAGENT or RESOURCES	SOURCE	IDENTIFIER
**Commercial assay kit**		
EasySep Human CD14+ isolation kit	STEMCELL technologies	Cat#19359
Mesenchymal stem cell growth medium (MSCGM™ BulletKit™)	LONZA	Cat# PT-3001
Human Mesenchymal Stem Cell (hMSC) Osteogenic Differentiation Medium BulletKit™	LONZA	Cat# PT-3002
Human IL-32 ELISA kit	R&D Systems	Cat# DY3040-05
Human TGF-β ELISA kit	R&D Systems	Cat# DY240-05
Human RANKL ELISA kit	R&D Systems	Cat# DY626
Human Osteoprotegerin (OPG) ELISA kit	R&D Systems	Cat# DY805
Human TRACP-5b ELISA kit	MyBioSource	Cat# MBS267836
**Software and algorithms**		
ZEN Microscopy Software	ZEISS Microscopy	
ImageJ software	FIJI	
FlowJo v10.6.2	BD Bioscience	
GraphPad Prism V9	GraphPad Software, LLC	

<u>Macro for TRACP Full Analysis Mean fluorescence intensity (MFI)</u>

```
ImageDir=getInfo("image.directory");

ImageName=getInfo("image.filename");

ImagePath=ImageDir+ImageName;

ImageNameNoExtension=File.getNameWithoutExtension(ImagePath);

print(ImageNameNoExtension)

run("Split Channels");
```

```
waitForUser("Close channels not needed");

run("Subtract Background...", "rolling=50");

run("Enhance Contrast", "saturated=0.35");

run("Duplicate...", " ");

//run("Threshold...");

run("Gaussian Blur...", "sigma=3");

setAutoThreshold("Otsu dark no-reset");

setOption("BlackBackground", false);

run("Convert to Mask");

run("Options...", "iterations=3 count=1 do=Open");

run("Create Selection");

roiManager("Add");

roiManager("save", ImageDir+ImageNameNoExtension+"_ROI.zip");

//calculate TRAcP MFI;

waitForUser("select TRAcP image");

run("Set Measurements...", "area mean display redirect=None decimal=1");

roiManager("Measure");

Area_TRAcP = getResult("Area",-1);

print("Area_TRAcP"+" "+Area_TRAcP);

MFI_TRAcP = getResult("Mean",-1);

print("MFI_TRAcP"+" "+MFI_TRAcP);

waitForUser("select dapi image");
```

```
// preprocess of the image

run("Subtract Background...", "rolling=50");

setOption("ScaleConversions", true);

run("8-bit");

run("Duplicate...", " ");

// Automatic threshold of the image

 setAutoThreshold("Triangle");

run("Convert to Mask");

run("Invert LUT");

run("Options...", "iterations=3 count=1 do=Open");

waitForUser("Select the TRAcP ROI in the ROI Manager");

run("Invert LUT");

run("Clear Outside");

// Separate touching nuclei

run("Invert LUT");

run("Watershed");

// count cells and eliminate too small-too big objects

run("Set Measurements...", "display redirect=None decimal=1");

run("Analyze Particles...", "size=20-700 show=[Overlay Masks] clear add");

number_of_cells = roiManager("count");

print("Number of cells"+" "+number_of_cells);

roiManager("Deselect");
```

```
roiManager("Delete");

run("Close All");
```

Macro for Osteocalcin Full Analysis MFI

```
ImageDir=getInfo("image.directory");

ImageName=getInfo("image.filename");

ImagePath=ImageDir+ImageName;

ImageNameNoExtension=File.getNameWithoutExtension(ImagePath);

print(ImageName)

run("Split Channels");

waitForUser("Close channels not needed");

run("Subtract Background...", "rolling=50");

run("Enhance Contrast", "saturated=0.35");

run("Duplicate...", " ");

//run("Threshold...");

setAutoThreshold("Yen dark no-reset");

setOption("BlackBackground", false);

run("Convert to Mask");

run("Options...", "iterations=1 count=1 do=Open");

run("Create Selection");

roiManager("Add");

roiManager("save", ImageDir+ImageNameNoExtension+"_ROI.zip");

//calculate Osteocalcin MFI;
```

```
waitForUser("select osteocalcin image");

run("Set Measurements...", "area mean display redirect=None decimal=3");

roiManager("Measure");

MFI_Osteocalcin = getResult("Mean",-1);

print(MFI_Osteocalcin);

waitForUser("select dapi image");

// preprocess of the image

run("Subtract Background...", "rolling=50");

setOption("ScaleConversions", true);

run("8-bit");

run("Duplicate...", " ");

//Automatic threshold of the image

setAutoThreshold("Triangle");

run("Convert to Mask");

run("Invert LUT");

run("Options...", "iterations=3 count=1 do=Open");

waitForUser("Select the Osteocalcin ROI in the ROI Manager");

run("Invert LUT");

run("Clear Outside");

// Separate touching nuclei

run("Invert LUT");

run("Watershed");
```

```
// count cells and eliminate too small-too big objects

 run("Set Measurements...", "display redirect=None decimal=3");

run("Analyze Particles...", "size=20-700 show=[Overlay Masks] clear add");

number_of_cells = roiManager("count");

print(number_of_cells);
```

Supplemental Figure 1

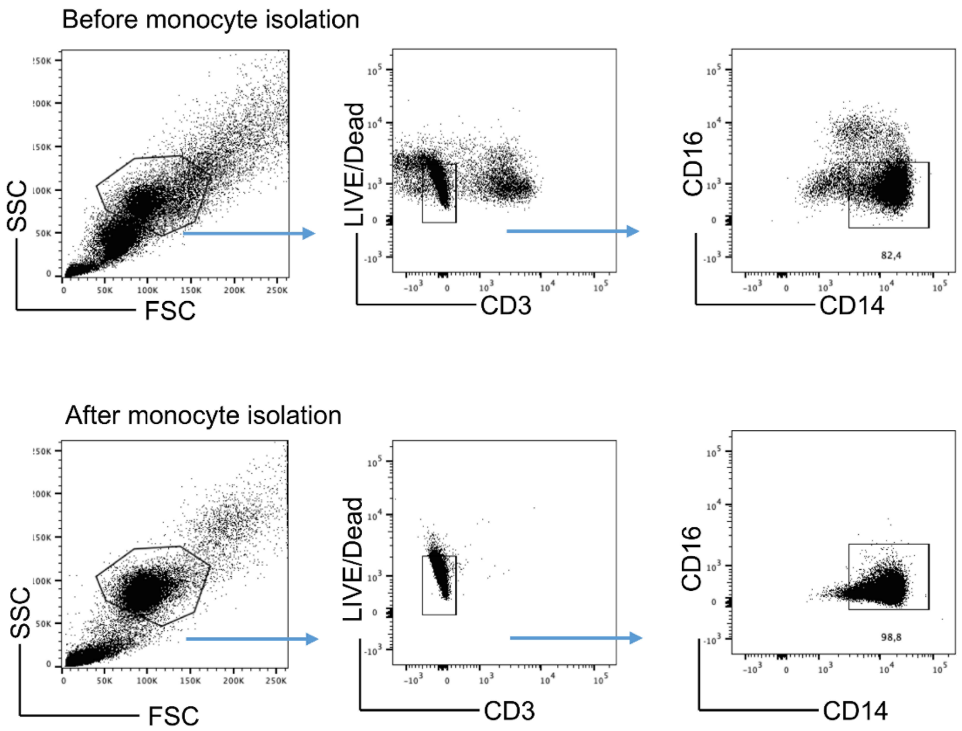

Figure S1: Flowcytometry Gating strategy on classical monocytes (CD14++CD16-). Representative monocyte isolation FACS blots showing classical monocytes gated on live CD3- subpopulation from total PBMCs followed by gating on CD14 and CD16 (upper panels). Lower

panel show the same gating on cells from the same donor after monocyte isolation using negative magnetic selection by EasySep Human CD14+ isolation kit from STEMCELL.

Supplemental Figure 2

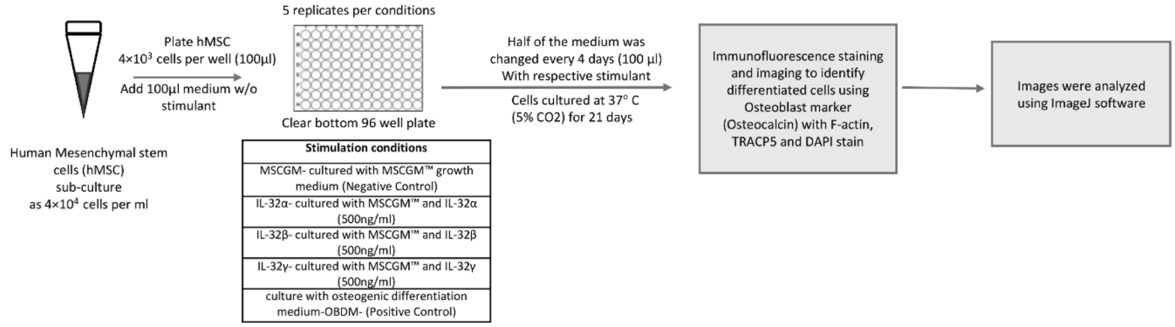

Figure S2: A diagram showing the differentiation process of human mesenchymal stem cells/hMSC in the presence or absence of IL-32 isoforms.

Supplemental Figure 3

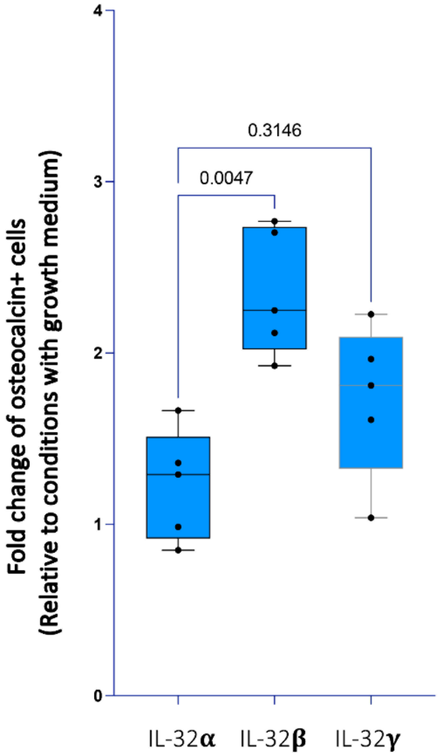

Figure S3: Comparison between the impact of individual IL-32 isoforms IL-32α, IL-32β, and IL-32γ on the differentiation of human mesenchymal stem cells (hMSCs) into osteoblasts (data normalized to conditions with growth medium and expressed as a fold change).

Supplemental Figure 4

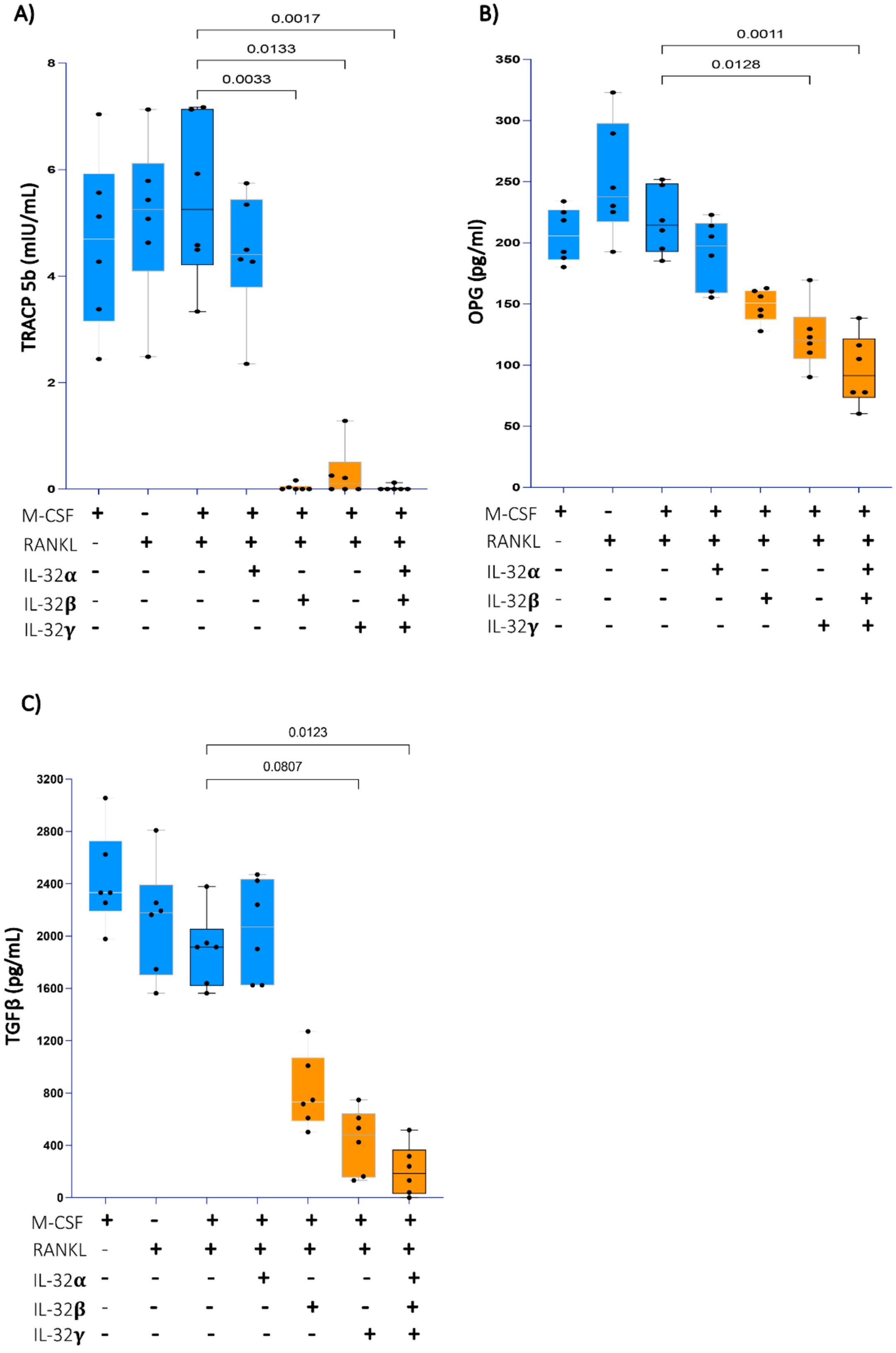

Figure S4: Impact of IL-32 isoforms on the expression of soluble osteoclast activation proteins. Soluble TRACP5b **(A)**, OPG **(B)** and TGF-β **(C)** measured in the supernatant of activated cells (monocytes isolated from n=6 donors) under the different stimulation conditions at the end of the incubation period of 21 days by ELISA. Nonparametric Kruskal-Wallis test and Dunn's subtests were performed for statistical analysis.

Supplemental Figure 5

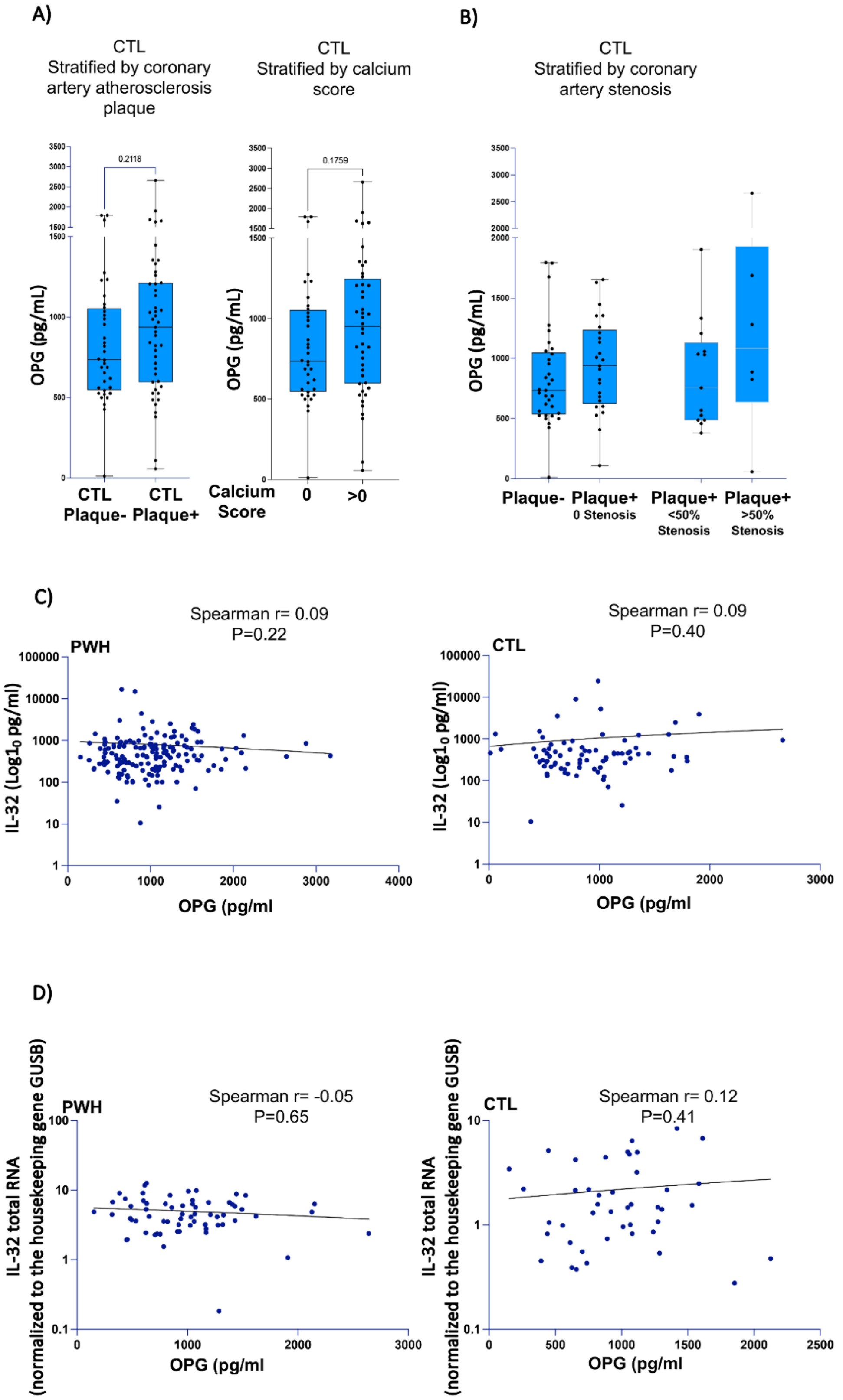

Figure S5: Plasma levels of OPG in control participants and correlations with IL-32. A) Left panel: Plasma collected from non-HIV controls (n=84) were used to measure OPG in participant with or without subclinical atherosclerotic plaques (plaque- n=35 and plaque+ n=47). Right panel: OPG levels on control participants after stratification by calcium score (available data on n=35 with calcium score 0 and n=44 with calcium score >0). **B)** OPG levels in plasma from participants for whom coronary artery maximum stenosis was calculated (n=34 with no coronary artery plaques, n=25 with measurable plaques but zero % stenosis, n=13 with measurable plaques and stenosis levels<50% and n=6 with measurable plaque and stenosis levels ≥50%). **C)** Correlation between plasma OPG levels and total IL-32 plasma protein from PWH (n=168, left panel) and controls (n=84, right panel). **D)** Correlation between plasma OPG levels and total cell-associated IL-32 RNA isolated from PWH (n=65, left panel) and controls (n=44, right panel). Data analyzed with the non-parametric Mann-Whitney test in **A**, Kruskal-Wallis and Dunn's sub test in **B** and Spearman correlations in **C**. CTL: Controls.
